# Mechanical coupling of supracellular stress amplification and tissue fluidization during exit from quiescence

**DOI:** 10.1073/pnas.2201328119

**Published:** 2022-08-01

**Authors:** Emma Lång, Christian Pedersen, Anna Lång, Pernille Blicher, Arne Klungland, Andreas Carlson, Stig Ove Bøe

**Affiliations:** ^a^Department of Microbiology, Oslo University Hospital, 0373 Oslo, Norway;; ^b^Department of Mathematics, Mechanics Division, University of Oslo, 0851 Oslo, Norway;; ^c^Department of Medical Biochemistry, Institute of Clinical Medicine, University of Oslo, 0372 Oslo, Norway;; ^d^Department of Biosciences, University of Oslo, 0371 Oslo, Norway

**Keywords:** quiescence, mechanical forces, collective migration, keratinocytes, epithelial monolayer

## Abstract

Cellular quiescence is a state of reversible cell cycle arrest that is associated with tissue dormancy. Timely regulated entry into and exit from quiescence is important for processes such as tissue homeostasis, tissue repair, stem cell maintenance, developmental processes, and immunity. However, little is known about processes that control the mechanical adaption to cell behavior changes during the transition from quiescence to proliferation. Here, we show that quiescent human keratinocyte monolayers sustain an actinomyosin-based system that facilitates global cell sheet displacements upon serum-stimulated exit from quiescence. Mechanistically, exposure of quiescent cells to serum-borne mitogens leads to rapid amplification of preexisting contractile sites, leading to a burst in monolayer tension that subsequently drives large-scale displacements of otherwise motility-restricted monolayers. The stress level after quiescence exit correlates with the level of quiescence depth at the time of activation, and a critical stress magnitude must be reached to overcome the cell sheet displacement barrier. The study shows that static quiescent cell monolayers are mechanically poised for motility, and it identifies global stress amplification as a mechanism for overcoming motility restrictions in confined confluent cell monolayers.

## Significance

Most cells in the human body reside in a dormant state characterized by slow growth and minimal motility. During episodes such as wound healing, stem cell activation, and cancer growth, cells adapt to a more dynamic behavior characterized by proliferation and migration. However, little is known about the mechanical forces controlling the transition from static to motile following exit from dormancy. We demonstrate that keratinocyte monolayers install a mechanical system during dormancy that produces a coordinated burst of intercellular mechanical tension only minutes after dormancy exit. The activated forces are essential for large-scale displacements of otherwise motility-restricted cell sheets. Thus, cells sustain a mechanical system during dormancy that idles in anticipation of cell cycle entry and prompt activation of motion.

Quiescence refers to a state of cell cycle arrest in which cells are retained in a standby mode, ready to re-enter the cell cycle upon activation by a given physiological stimuli. The pool of quiescent cells in the human body is typically represented by tissue-specific stem and progenitor cells, naive immune cells, fibroblasts, and epithelial cells (1, 2). In addition, certain cancer cells have the ability to evade cancer therapy by entering a dormant quiescence-like state (1, 2). Accordingly, careful regulation of entry into and exit out of quiescence is important for several physiological processes such as tissue homeostasis and repair, stem cell maintenance, immunity, reproduction, and development (1, 2).

During homeostasis, the balance between quiescent and proliferating cells is controlled by constituents of the microenvironment such as soluble factors, extracellular matrix components, blood vessels, and neighboring cells. On the other hand, during episodes that require extensive tissue renewal and remodeling, for example after injury, coordinated stimulation of quiescent cells into proliferation is facilitated by increased exposure to blood-borne and cell-secreted mitogens through local inflammatory responses such as increased blood flow, increased vascular permeability (vasodilation), and immune cell recruitment (3, 4). Accordingly, a commonly used methodology for studies of quiescence in cultured mammalian cells involves consecutive treatments with serum-free and serum-containing growth medium (1).

Quiescent cells are required to maintain a high level of preparedness in order to facilitate rapid activation of specialized cell functions once cell division is stimulated. In agreement with this, quiescent stem cells and naive immune cells have been shown to possess multiple epigenetic and posttranslation mechanisms that facilitate the rapid expression of linage-specific genes following stimulation of quiescence exit (2, 5–14). However, little is known about mechanical forces that facilitate adaptation to cell cycle–activated behaviors.

Quiescence exit is frequently associated with activation of cell motility. For example, quiescent stem and naive immune cells migrate out of their niches in response to cell cycle activation in order to support tissue homeostasis, repopulate injured tissue, or to perform immune surveillance at distal locations (15–18). In addition, reawakening of dormant quiescent cancer cells can cause tumor relapse and formation of metastases years after remission (19). In multilayered epithelial tissue, like the skin, exit from quiescence during homeostasis is associated with lateral migration to suprabasal regions, while skin injury evokes massive reawakening of basally localized keratinocytes concomitant with activation of cell sheet displacement by collective migration to restore damaged epidermal surfaces (20–23). The strong correlation between quiescence exit and cell migration in multiple physiological settings suggests the existence of mechanisms that link quiescence exit to activation of cell motility.

The dynamics of epithelial collectives is largely regulated by mechanical forces generated through cell–cell interactions as well as interactions between cells and the extracellular environment (24). Key components involved in controlling these forces are cytoskeletal components such as actinomyosin and adhesion complexes such as adherent junctions and focal adhesion complexes (25). Additional factors that have been reported to influence the dynamic behavior of epithelial monolayers include the presence of epithelial edges (24, 26), mechanical stretching or compression (27, 28), expression of the endosomal Rab5 protein (29), exposure of cells to growth factors (30–32), local changes in cell shape (33), and the ability of cells to undergo neighbor exchange (34, 35). In addition, recent studies have also identified a functional link between cell cycle progression and force fluctuation leading to dynamic behavior of cultured epithelial monolayers (36, 37).

In this study, we have investigated a mechanical link between quiescence exit and activation of large-scale cell sheet displacements. Using traction force microscopy (TFM), we found that confluent cell monolayers install an actinomyosin-based system during quiescence that produces a coordinated burst of contractile forces and intercellular tension across the epithelial monolayer immediately following exposure to serum-borne mitogens. By combining experiments and theoretical modeling, we show that the amplified forces are essential for driving coordinated cell sheet displacements within otherwise motility-restricted cell monolayers. Furthermore, the magnitude of mechanical forces created during quiescence exit and the extent of cell sheet displacement correlate with quiescence depth. Our study provides evidence that quiescent keratinocyte monolayers possess mechanical preparedness for motility and establish monolayer stress amplification as a strategy for overcoming the motility barrier in confined cell sheets.

## Results

### Quiescence-Dependent Cell Sheet Displacements Emerge through Collective Migration and Large-Scale Deformations.

**Fig. 1.**
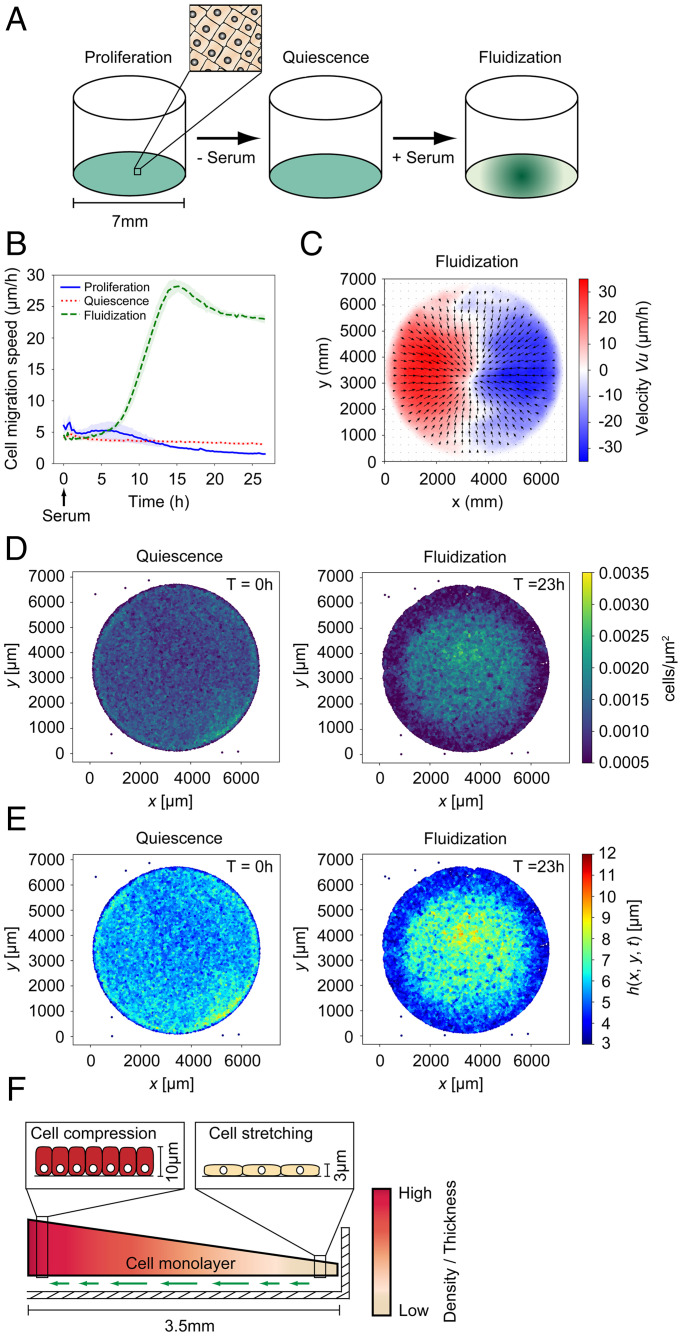
Quiescence-dependent cell sheet displacements by collective migration and large-scale deformation. (*A*) Schematics of the methodology used for the generation of quiescence-dependent cell sheet displacement. (*B*) Cell migration speed in confluent monolayers without treatment (proliferation), monolayers subjected to serum depletion for 48 h (quiescence), and monolayers subjected to serum depletion for 48 h followed by serum restimulation (fluidization). Data are based on PIV analysis and show average migration speed across the monolayer surface ± SD; *n* = 8 separate monolayers per sample. See also Video S1. (*C*) PIV-derived vector fields 18 h after serum restimulation. Average values of eight separate monolayers are shown. The color bar displays the x component of the velocity field. (*D*) Cell density at 0 h and 23 h after serum restimulation. See also Video S2. (*E*) Cell sheet thickness at 0 h and 23 h after serum restimulation. See also Video S2. (*F*) Schematic showing cell sheet displacement (green arrows) and deformation through a coordinated flow of cells. The cross section represents one-half (3.5 mm) of the confluent cell sheet in a well of a 96-well plate.

In a previous study, quiescent HaCaT keratinocytes were observed to transform from static to motile cell sheets following exposure to fetal bovine serum (FBS). The serum-activated motions observed in these experiments are coordinated over length scales exceeding several millimeters and depend on prior activation of quiescence through serum depletion (31). In the present study, we imaged mCherry-tagged nuclei within HaCaT keratinocyte monolayers seeded in 96-well plates using an automated high-content imaging system. Using this approach, we were able to analyze cell sheet velocity, cell density, and cell sheet thickness based on multiple cell monolayer samples confined to a defined circular surface (7.0 mm in diameter) (Fig. 1*A*). We observed limited cell motion within confluent cell monolayers that had been plated in serum-containing medium 20 h prior to imaging and cell monolayers that had been subjected to an additional 2 d of serum depletion prior to imaging (Fig. 1*B* and Video S1). This is consistent with previous reports showing that epithelial cells undergo jamming and immobilization at high densities when confined to a fixed area (38, 39). In contrast, serum reexposure of serum-depleted monolayers led to quiescence-dependent cell sheet displacements (Fig. 1*B* and Video S1).

The serum-activated quiescent monolayers exhibited a remarkably uniform cell migration pattern within the 96-well plate that was characterized by a collective flow of material away from the monolayer edges toward the central region of the well (Fig. 1 *C*–*E* and Video S2). An analysis of cell density revealed a progressive increase in cell density at the center concomitant with decreasing cell density near the monolayer edges (Fig. 1*D* and Video S2). To analyze monolayer compression and extension, we developed an algorithm for mapping the monolayer thickness based on its cellular density (*SI Appendix*, Fig. 1). Mapping of monolayer thickness in serum-activated quiescent cell sheets revealed a progressive increase in thickness at monolayer centers (maximum cell sheet height of 10 µm) and a compensatory decrease in monolayer thickness at the cell sheet edges (minimal cell sheet height of 3 µm) following serum-induced exit from quiescence (Fig. 1*E* and Video S2). We also investigated a potential remodeling of actin at the basal cell surface using live confocal microscopy of LifeAct-expressing HaCaT cells. We observed a rapid disappearance of stationary actin-enriched foci at the basal side during the first 3 h after serum restimulation (Video S3). This was followed by the appearance of dynamic actin-enriched feet that coincides with the activation of cell sheet displacement 5 to 8 h after serum stimulation (Video S4). Finally, cells did not exhibit extensive neighbor exchange within the fluidized collectives as determined by the inspection of local groups of 20 to 40 cells (Video S5). Thus, the cells migrate in a highly organized fashion with limited positional exchange. Combined, these results show that serum-activated quiescent cell sheets mediate large-scale displacements through collective cell migration and redistribution of mass (Fig. 1*F*).

### Serum Activation of Quiescent Cells Generates an Immediate Burst of Traction Forces and Intercellular Stress through Amplification of Preexisting Contractile Sites.

**Fig. 2.**
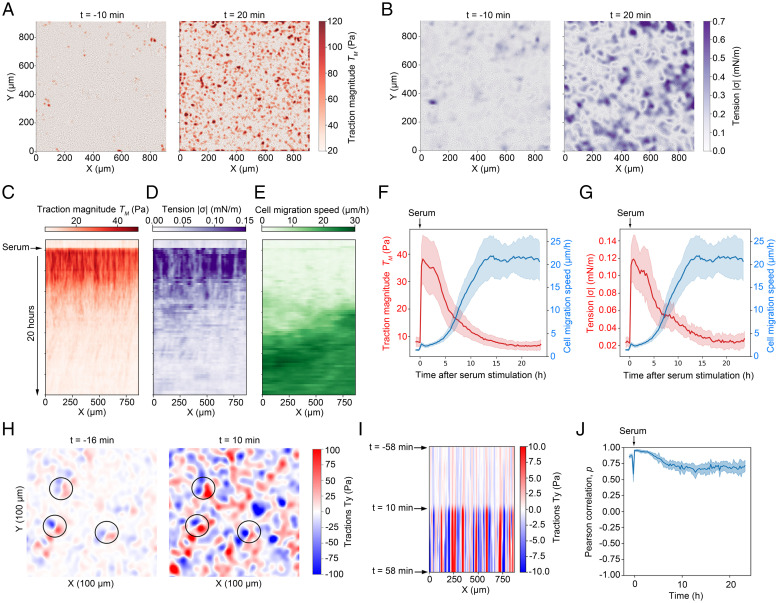
Serum activation of quiescent monolayers is accompanied by activation of global stress through rapid amplification of preexisting stress centers. (*A*) Traction force maps generated 10 min before (*Left* panel) and 20 min after (*Right* panel) serum activation of serum-depleted monolayers. See also Video S6. (*B*) Tension maps 10 min before (*Left* panel) and 20 min after (*Right* panel) serum activation of serum-depleted monolayers. See also Video S6. (*C*–*E*) Kymographs showing traction magnitude (*C*), intercellular tension (*D*), and migration speed (*E*) before and after serum stimulation of a confluent quiescent cell sheet. A representative microscopic field 48 min before and 20 h after serum stimulation is shown. (*F*–*G*) Traction magnitude (*F*) and intercellular tension (*G*) relative to cell migration speed over time. The plotted lines show average ± SD; *n* = 6 separate microscopic fields. (*H*–*J*) Preexisting local tractions in quiescent cell sheets are amplified by serum restimulation. (*H*) Map showing *T_y_* traction force component 16 min before (*Left* panel) and 10 min after (*Right* panel) serum restimulation. Examples of amplified traction sites are highlighted by circles. (*I*) Kymograph displaying the traction force *T_y_* component before and after serum restimulation of a confluent quiescent cell sheet. (*J*) Plot showing time evolution of the Pearson correlation coefficient. Each of the plotted values represents the Pearson correlation between adjacent frames. *n* = 6 separate microscopic fields.

To investigate the mechanical forces that regulate quiescence-dependent collective cell migration, we extracted traction forces and the intercellular stress in cell monolayers using TFM. To achieve this, we plated HaCaT keratinocytes on a soft (4 kPa) substrate consisting of collagen IV-coated polyacrylamide containing fluorescently labeled beads as fiducial markers. We analyzed multiple microscopy fields (1.3 × 1.3 mm each) within 12-well glass bottom plates. Cell sheets plated on soft acrylamide substrates exhibited a quiescence-dependent migration phenotype similar to that observed for cells grown on a glass surface. We noted a lower maximal migration speed for cells plated on acrylamide (15 to 20 µm/h) compared to cells plated on glass (25 to 35 µm/h), a difference that could be attributed to differences in substrate softness. An analysis of cell-mediated substrate displacements at time points before and after serum stimulation revealed an instant increase in traction forces and intercellular stresses across the entire confluent cell sheet after serum stimulation (Fig. 2 *A* and *B* and Video S6). This burst in mechanical forces peaked between 10 and 30 minutes (min) after serum exposure and progressively decayed for a period of more than 20 h concomitant with the activation of cell sheet motility (Fig. 2 *C*–*G*). The inverse correlation between monolayer tension and cell sheet displacement activity, after an initial burst in traction force magnitude, was unexpected since most studies performed on single cells and cell collectives suggest a positive correlation between traction forces and cell motility (40–43).

We next analyzed the relative positions of traction force centers before and after serum stimulation. We found that local serum-stimulated tractions frequently arise from weaker tractions already present in the quiescent unstimulated cell sheet (Fig. 2 *H* and *I*). To quantify the correlation between force-generating sites before and after serum stimulation, we used the Pearson correlation coefficient. For this parameter, the absence of a correlation is represented by 0, while 1 and −1 represent perfect correlation and anticorrelation, respectively. Calculation of the Pearson correlation coefficient between consecutive force maps in a time series of serum-stimulated monolayers revealed a significant positive correlation of force distribution before and after serum stimulation (Fig. 2*J*). This result shows that quiescent monolayers are poised for a high global stress by means of numerous weak local tractions that can be amplified upon exposure to serum-borne growth factors.

We also analyzed the individual traction force components in x-direction (*T_x_*) and in y-direction (*T_y_*) within microscopic fields with the aim of identifying potential biases in traction force orientation. Inspection of the traction components did not reveal a preferential direction of force orientation across larger microscopy areas (*SI Appendix*, Fig. 2 *A* and *B*). In addition, plotting the average of the traction force component *T_x_* against an increasing microscopic field size suggested that the average traction forces in the x or y direction quickly approached zero for microscopic fields larger than 100 × 100 µm (*SI Appendix*, Fig. 2*C*). Thus, the orientation of local traction forces before and after serum stimulation is largely random relative to the direction of subsequent cell sheet displacement.

### Stress Amplification Is Actinomyosin-Dependent and Coupled to Cell Sheet Displacement.

**Fig. 3.**
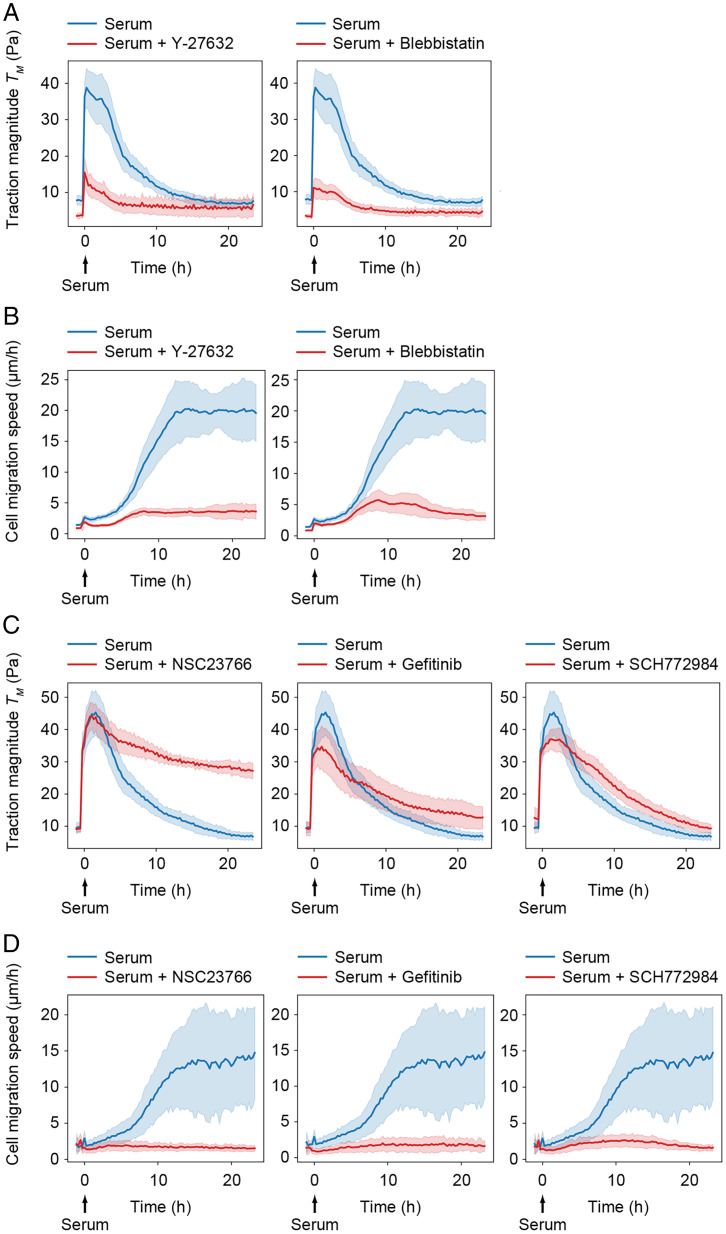
Dependency between global stress amplification and cell sheet displacement. (*A*) Traction force magnitudes and (*B*) cell migration speed during serum activation in the presence of the actinomyosin inhibitors Y-27632 or blebbistatin. (*C*) Traction force magnitudes and (*D*) cell migration speed generated in the presence of collective cell migration inhibitors specific for Rac1 (NSC23766), EGFR (Gefitinib), and ERK (SCH772984). (*A*–*D*) The time of serum stimulation (t = 0) is indicated. All graphs show average values ± SD from eight separate microscopy fields.

Actinomyosin is known to regulate tractions and stress in cells during single cell or collective cell migration. To investigate the role of actinomyosin in quiescence-dependent collective migration, we serum activated quiescent cell sheets grown on soft acrylamide substrates in the presence or absence of the Rho-associated kinase inhibitor Y-27632, the nonmuscle myosin inhibitor blebbistatin, the actin polymerization inhibitor latrunculin A, and the formin inhibitor SMIFH2. The presence of these inhibitors led to a significant reduction in traction force amplification and cell sheet velocity after serum activation (Fig. 3 *A* and *B* and *SI Appendix*, Fig. 3 *A* and *B*).

We next examined a potential link between the amplified mechanical forces and cell migration by performing pharmacological inhibition of Rac1, EGFR, and ERK, which are protein components that have been reported to play critical roles in mediating collective cell migration in epithelial monolayers (31, 44). We also included in this experiment two distinct inhibitors with specificity for focal adhesion kinase (FAK) to perturb a potential role of integrin signaling pathways in cell migration. The inhibitors abrogated cell migration and caused a concomitant reduction in traction force decay rates after the initial serum-mediated traction force amplification (Fig. 3 *C* and *D* and *SI Appendix*, Fig. 3 *C* and *D*). For cell cultures treated with Rac1 or FAK inhibitors, the traction force amplification after serum stimulation seemed unaffected, while traction force decay rates were reduced compared to that of control-treated cells (Fig. 3*C* and *SI Appendix*, Fig. 3*C*). For cells treated with EGFR or ERK inhibitors, the effect on traction force decay was significant but less pronounced, possibly due to a dual role of these inhibitors in interfering with both traction force amplification and cell migration (Fig. 3*C*). These observations indicate that exposure of quiescent monolayers to serum stimulates actinomyosin-mediated monolayer tension and that the subsequent large-scale cell sheet displacements are driven by monolayer tension decay.

### Theory Predicts Serum-Induced Stress Amplification as a Key Component for Global Cell Sheet Displacement.

**Fig. 4.**
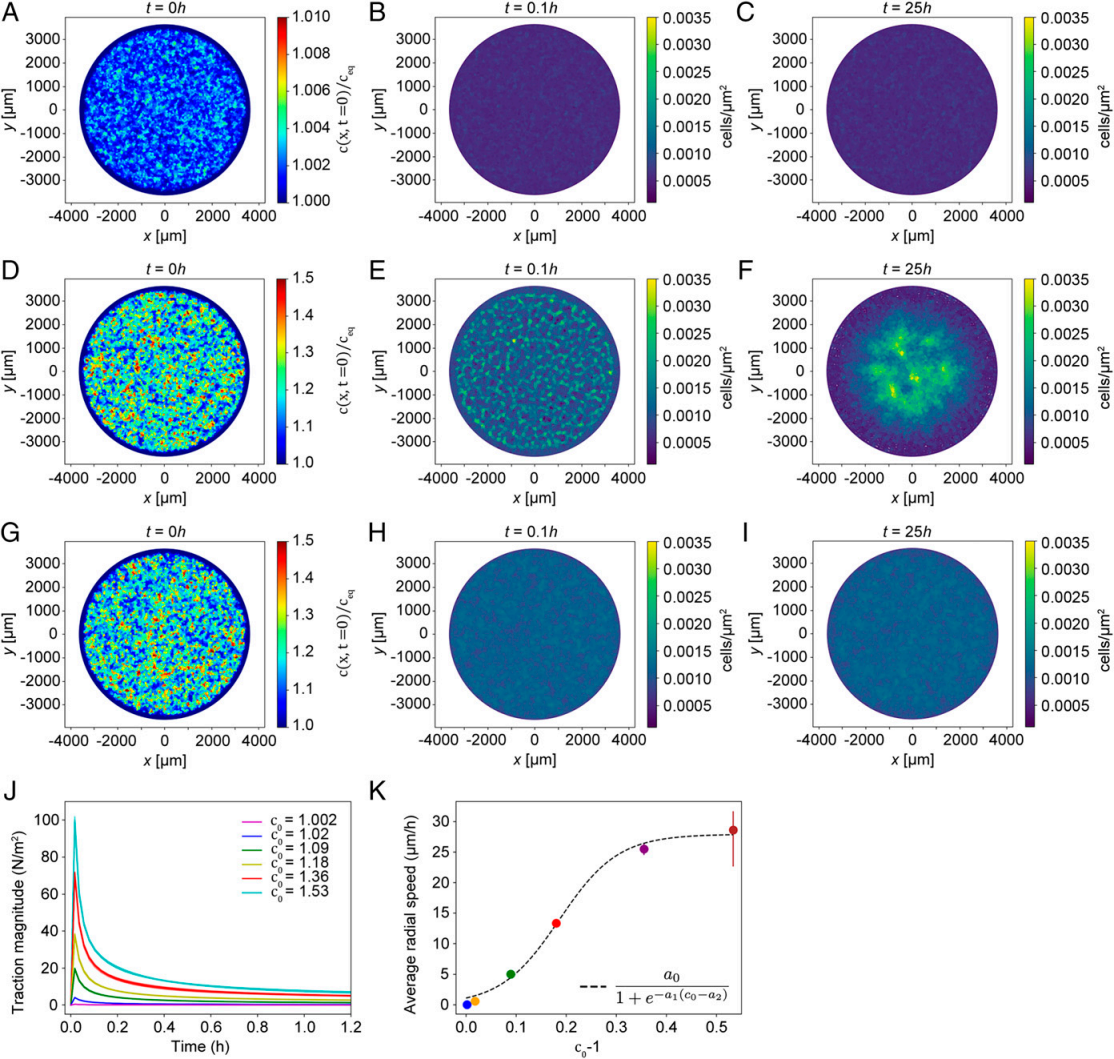
Theory predicts serum-induced stress amplification as a key component for global cell sheet displacement. (*A*) Normalized initial concentration $c(x,t=0)/c_{eq}$ used to calculate the results shown in *B* and *C* with *c_0_* =1.002, see also Video S7. (*B*) Monolayer displacement field ***u***(***x***, *t*) at time t = 0.1 h. Small local contractions are formed at points that have a high initial concentration. (*C*) Monolayer displacement field ***u***(***x***, *t*) at time t = 25 h. At long times, the small contractions have disappeared, and no global contraction of the cell sheet is formed. (*D*) Normalized initial concentration $c(x,t=0)/c_{eq}$ used to calculate the results shown in panels *E* and *F* with *c_0_* =1.2, see also Video S8. (*E*) Monolayer displacement field ***u***(***x***, *t*) at time t = 0.1 h. Local contraction centers are formed at points that have a high initial concentration and gradients. (*F*) Monolayer displacement field ***u***(***x***, *t*) at time t = 25 h. At long times, we observe a collective migration toward the center of the monolayer, forming one large central contraction center. (*G*) Normalized initial concentration $c(x,t=0)/c_{eq}$ used to calculate the results shown in *H* and *I* with *c_0_* =1.2 and a reduced value of α compared to *A*–*F*. (*H*) Monolayer displacement field ***u***(***x***, *t*) at time t = 0.1h. (*I*) Monolayer displacement field ***u***(***x***, *t*) at time t = 25 h. Reducing the contractile strength α inhibits displacements and the motion of the cell sheet. (*J*) The traction magnitude is shown as a function of time for different levels of prestress *c_0_*. (*K*) The average radial velocity extracted from the numerical simulations. The dashed line is a fitted sigmoid function $a_{0}/(1+{\text{e}}^{-a_{1}\left(c_{0}-a_{2}\right)})$ with coefficients $a_{0}=\text{maximal}\;\text{speed}=28\;\;\mu \text{m}/h,a_{1}=17.1,a_{2}=0.19$. The parameters fixed in the numerical simulations shown in the main text and in *A*–*K* are E = 4,000 Pa, h_eq_ = 8 μm, D = 10^−9^ m^2^ s^−1^, Γ = 2.5⋅10^8^ Ns m^−3^, β/c_eq_ = 8 × 10^−5^ s^−1^, and τ_c_ = 13,000 s which are further described in *SI Appendix*.

To understand how the stress distribution in the cell sheet can lead to collective cell migration, we developed a minimal mathematical model based on a phenomenological active gel theory (45–51) (*Materials and Methods* and *SI Appendix*). The model encompasses the most essential physical components when treating the cell layer as a continuous field, i.e., elastic deformations, cell–substrate friction, and viscous friction, in addition to an active stress induced by the actinomyosin (described by the field c(x,t)). The initial experimental conditions were mimicked in the numerical simulations when solving the mathematical model; we used a circular domain, with an initial random distribution of the actinomyosin giving the prestressed state of the sheet, i.e., $c_{0}=\frac{\sum^{\text{​}}c(x,t=0)dA}{c_{\text{eq}}\cdot A}$_,_ with *dA* the area of the triangulated mesh, *A* the area of the domain and $c_{\text{eq}}$ the equilibrium cell concentration (*Materials and Methods*). At low level of local contractility, we observed little net motion and a global cell sheet displacement failed to appear (Fig. 4 *A*–*C* and Video S7). We then tested in numerical simulations how the increased level of prestress $c_{0}$ affects the cell sheet dynamics by systematically increasing its magnitude. We found that an increase in monolayer prestress correlates with increased activation of radial displacement from the edges of the monolayer toward the center (Fig. 4 *D*–*F* and Video S8). Thus, at sufficiently high prestress levels, the theoretical model recapitulates the dynamic behavior observed in serum-stimulated quiescent cell monolayers. To further verify the model, we performed numerical simulations using low values of $\hat{\alpha }$, the parameter that sets the magnitude of actinomyosin contractility. We found that a reduced value of $\hat{\alpha }$ abrogates the activation of traction forces (*SI Appendix*, Fig. 4) and cell sheet displacement (Fig. 4 *G*–*I*). Thus, the simulation results are in agreement with our experimental observations showing that actinomyosin is required for force generation and motility.

**Fig. 5.**
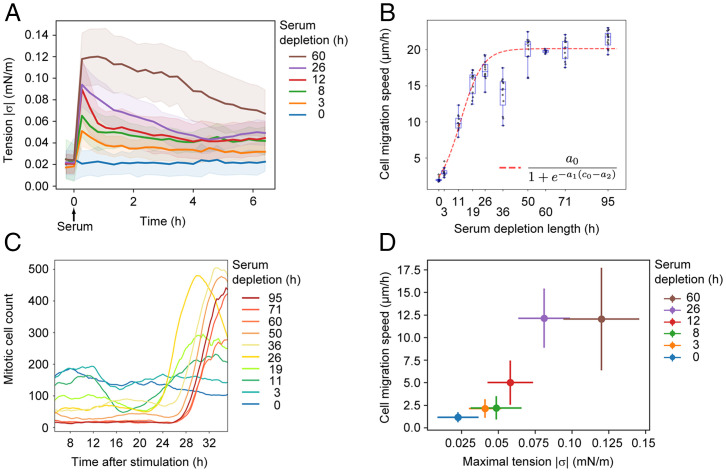
The magnitude of stress amplification correlates with quiescence depth. (*A*) Time evolution of intercellular tension produced after different time periods of serum depletion. Graph shows average values ± SD; *n* = 10 to 25 separate microscopic fields from two independent experiments. Time of serum activation (t = 0) is indicated. (*B*) The effect of serum deprivation length on cell migration. Monolayers in 96-well plates were subjected to serum deprivation for the time periods indicated and subsequently restimulated with serum. Migration speed is plotted as the mean velocity between 8 and 50 h after serum restimulation. *n* = 8 to 16 separate microscopic fields from two independent experiments. The dashed line is the curve extracted from the theoretical predictions $a_{0}/(1+{\text{e}}^{-a_{1}\left(c_{0}-a_{2}\right)})$, (see Fig. 4*K*), but substituting $c_{0}$ with the serum depletion time. The coefficients are $a_{0}=\text{maximal}\;\text{speed}=20µ\text{m}/\text{h},\;a_{1}=0.16,a_{2}=12.2$. (*C*) Estimation of the cell division frequency after different lengths of serum depletion prior to serum restimulation using an automated detection of mitotic cells. Graph shows the moving average of mitotic cell counts and is representative of two independent experiments. (*D*) Plot showing cell sheet displacement magnitude versus intercellular tension. Maximal tension is represented by the average tension measured at 1 h after serum restimulation. Average speed is represented by the average speed of all time points between 8 and 20 h after serum restimulation. The plot uses the same set of raw data as for *A*.

To further quantify the influence of the prestress, we extracted the averaged traction force (Fig. 4*J*) and the averaged radial velocity (Fig. 4*K*) for the different initial magnitudes of c(x,t=0) in the numerical simulations. We observed a steep increase in the traction force magnitude that decays with time (Fig. 4*J*). Furthermore, the radial velocity scale with the magnitude of initial stress until a threshold value of the active actinomyosin was approached whereby the velocity stagnated (Fig. 4*K*). An almost linear response in radial velocity with respect to the magnitude of c(x,t=0) and the length over which c(x,t=0) varies was predicted from a scaling analysis of the mathematical model (*SI Appendix*, Fig. 5). However, as the cellular density at the center of the monolayer increases, the elastic compression will balance the concentration gradient and thus lead to a stagnation and subsequent reduction in both c(x,t) and the cell average radial velocity. The form of the curve passing through the values from the simulations can be captured with a sigmoid function, $a_{0}/(1+{\text{e}}^{-a_{1}\left(c_{0}-a_{2}\right)})$, where $a_{0}$ is set by the speed of the sheet as it saturates for large values of the prestress and the two parameters a_1_ and a_2_ describe the shape of the curve and are found by fitting the data from the numerical simulations (Fig. 4*K*).

### The Magnitude of Serum-Stimulated Stress Amplification and Cell Monolayer Displacement Correlates with Quiescence Depth.

To experimentally test the theoretical predictions that the initial stress level regulates cell sheet displacement dynamics, we reasoned that the monolayer stress can be tuned by adjusting the length of the serum deprivation period prior to serum reexposure. We therefore examined the relative relationship between the serum depletion length and the level of intercellular tension amplified after serum stimulation. We found that the serum depletion length correlates with the level of intercellular stress produced upon serum restimulation (Fig. 5*A*). We next measured the velocity in serum-exposed cell sheets that had been subjected to different serum starvation length periods. By plotting these values against a normalized time axis representing starvation length together with the sigmoid function representing the theoretical predictions of prestress versus velocity (Fig. 4*K*), we observed good agreement between the experimental observations and the phenomenological model (Fig. 5*B* and *SI Appendix*, Fig. 6). To analyze the effect of serum depletion length on quiescence depth, we monitored the surge of cell divisions that occur between 24 and 30 h after the reexposure of quiescent cells to serum. We found that the serum deprivation length correlates with the time required for cells to enter mitosis after serum reexposure (Fig. 5*C*). This suggests that the level of monolayer stress after serum restimulation correlates with quiescence depth. Finally, by plotting tension values measured in serum-stimulated monolayers subjected to different serum depletion lengths against cell sheet velocities, we found that a threshold value of tension between 0.05 and 0.12 mN/m is required in order to achieve effective collective migration (Fig. 5*D*). Together, predictions from a minimal theoretical model and experimental observations suggest that the transition of quiescent monolayers from immobile to dynamic motile cell sheets is mediated through rapid and coordinated amplification of numerous traction force centers dispersed throughout the monolayer. Indeed, both the theoretical model and the experiments show that a monolayer has to generate a critical level of global stress in order to achieve effective coordinated dynamic behaviors.

## Discussion

Many biological processes, including developmental processes, wound healing, and activation of dormant cancer cells, involve cell cycle re-entry concomitant with activation of increased cell motility and tissue plasticity. In agreement with this, the present study identifies a mechanism responsible for the dynamic behavior of confluent human keratinocyte collectives following exit from quiescence. Activation of the forces leading to the dynamic behaviors occurs only minutes after exposure to serum-borne mitogens, and the magnitude of forces and monolayer displacement activity correlate with quiescence depth (duration of the quiescence period). These observations argue that the process of quiescence entry and exit should be viewed as a cycle of events, where cellular processes required during departure from quiescence are mechanically prepared during quiescence entry. A similar dependency between events taking place before and after exit from quiescence has previously been reported to facilitate linage-dependent gene expression following activation of quiescent stem cells and naive immune cells (2, 5–14). Thus, our observation that quiescent epithelial monolayers are prepared for motility is consistent with the emergent view that quiescence represents a poised cell state, where multiple cellular processes have been prepared to facilitate activities required during episodes of cell cycle entry. Given the close connection between cell cycle activation and activation of cell motility, it will be important to determine a potential role of bona fide cell cycle drivers such as cyclin-dependent kinases (CDKs), CDK inhibitors, retinoblastoma, and E2F in quiescence-dependent cell migration—a possibility which is currently under investigation.

At least two distinct mechanisms have previously been reported to be responsible for the solid-like to fluid-like transition of confined epithelial collectives. The most studied of these mechanisms is the epithelial-to-mesenchymal transition (EMT), which is characterized by the deconstruction of intercellular adhesion complexes, disruption of the epithelial permeability barrier, and formation of elongated cells with front–rear polarity that promotes directed cell migration and invasion (52). The other mechanism is commonly referred to as an unjamming transition (UJT) and is associated with activation of large-scale coordinated collective migration and local cell shape remodeling (29, 33). The dynamic behaviors of keratinocyte collectives following serum-activated exit from quiescence appear to exhibit some features associated with the UJT. For example, both UJT and serum-activated keratinocytes have been shown to be dependent on EGFR-mediated signaling, and in both cases, highly coordinated cell displacements are observed without dramatic impairment of epithelial integrity (27, 31, 53). On the other hand, serum-activated keratinocytes have been shown to generate a front–rear polarized organization of organelles during migration, consistent with the emergence of cellular front–rear polarity during EMT (31). Further investigations should aim to characterize both differences and similarities between distinct epithelial fluidization mechanisms. It will also be important to identify the specific roles played by each of the mechanisms in biological functions and during pathogenesis.

The large-scale features of the cell sheet, which extends across a circular domain with a radius of ∼7 mm and contains between 150,000 and 200,000 cells, have been described by a phenomenological continuum model treating the cell sheet as an active gel. Capturing the dynamics of such a large number of cells with a cell-based model would be challenging. However, an interesting avenue to explore further is to also look into the details of parts of the cell layer and how the individual cell dynamics after serum stimulation are affected by, e.g., cell-to-cell junction fluctuations, formation of topological defects, turnover of adhesion proteins during migration, cell shape dynamics, cell cycle regulation, epithelial monolayer jamming, and UJTs, to determine how these small-scale effects couple to the large-scale collective cell migration we observe.

Both the experiments and the numerical simulations presented in the present study point to a key role of amplified traction forces and supracellular tension in mediating the transition from static to dynamic behavior. The requirement of a critical stress level for activation of tissue motility is consistent with previous studies showing that motion in developing tissues is driven by supracellular stress fluctuations (54, 55). In addition, intercellular stress has been shown to contribute to the invasive behavior of epithelial edges (24, 56) as well as local cell–cell junction deformations (36). From an evolutionary perspective, it may be energetically favorable for organisms to maintain a stable but low prestress in epithelial tissues during periods of tissue dormancy. The ability to mediate a coordinated burst of stress distribution in response to serum-borne mitogens may have evolved to support the rapid onset of coherent cell motion during events that require tissue remodeling.

## Materials and Methods

### Cells.

The immortalized human keratinocyte cell line HaCaT (57) and its derivatives were grown in Iscove’s modified Dulbecco’s medium (IMDM; MedProbe) supplemented with 10% FBS (Thermo Fisher Scientific) and 90 U/mL penicillin/streptomycin (PenStrep; Lonza). For serum depletion and serum reactivation, we used the same medium, but with 0 and 15% FBS, respectively. HaCaT cells expressing mCherry-tagged Histone H2B has been described previously (31). HaCaT cells stably expressing LifeAct-RFP were produced by using recombinant lentivirus particles obtained from Ibidi.

### Drug Treatment.

Inhibitors were used in the study at the following concentrations: 7.8 µM Y-27632 (Y0503), 6.8 µM blebbistatin (B0560), 5 µM gefitinib (Y0001813), 100 µM NSC23766 (SML0952), 1.19 µM latrunculin A (428021), and 20 µM SMIFH2 (s4826) purchased from Merck and 10 µM SCH7772984 (S7101), 5 µM TAE226 (S2820), and 5 µM PF562271 (S2890) purchased from Selleck Chemicals.

Cells that had undergone serum depletion for 48 h were pretreated with drugs for 1 h in serum-depleted medium before they were placed under the microscope. Images of cells and polyacrylamide-embedded beads were then acquired at three time points (intervals of 16 min) before serum activation by adding 1 volume of medium containing 30% FBS and 2× concentrations of respective drugs. Control cells were treated the same but without drugs.

### High-Content Imaging of Monolayers Confined to the Bottom Surface of 96-Well Plates.

HaCaT mCherry-Histone H2B cells were seeded in 96-well glass bottom plates (Greiner Sensoplate [M4187-16EA, Merck]) at 75,000 cells per well. Cells were consistently seeded 18 to 22 h prior to serum depletion or image acquisition (also when different lengths of serum depletion were compared). Live-cell imaging of monolayers was performed using the ImageXpress micro confocal high-content microscope controlled by the MetaXpress 6 software (Molecular Devices). Image acquisition was carried out in widefield mode using a 4× 0.2 numerical aperture (NA) PLAPO air objective at pixel binning 2, filter set for detection of mCherry fluorescence, and an environmental control gasket that maintain 37 °C and 5% CO_2_. Four tiled images per well (covering the entire well surface) were acquired for a total time period of 30 to 50 h using a time interval of 16 min between frames. Acquired time lapse movies were then processed by tiling (using the MetaXpress software) to generate movies covering the bottom surface of the entire well.

### Preparation of PAA Gel Substrates.

Collagen IV–coated polyacrylamide (PAA) gels were prepared in 12-well glass bottom multiwell plates (P12G-1.5-14-F; MatTek Corporation) using a protocol modified from Serra-Picamal et al. (58). The glass surfaces were activated by adding Bind-silane (GE17-1330-01; Merck): MQ-H_2_O (Milli-Q Reference Water Purification System): acetic acid at a 1:12:1 ratio for 4 min followed by two quick washes in 1× phosphate buffered saline (PBS). Next, polyacrylamide gels (4.0 kPa) were prepared by mixing a 93.75 µL 40% (7.5%) acrylamide solution (1610140; Bio-Rad), 37.5 µL 2% Bis-solution (161-0142; Bio-Rad), 2 µL (0.4%) carboxylate-modified fluorescent beads (FluoSpheres carboxylate-modified, 0.2 µm, 580/605; F8810; Thermo Fisher Scientific), 2.5 µL ammonium persulfate (0.05%; Bio-Rad), 0.25 µL tetramethylethylenediamine (TEMED) (0.05%; 161-0801; Bio-Rad), and 364 µL MQ-H_2_O. A total of a 10 µL freshly prepared gel solution was placed as droplets at the bottom of each glass bottom well and subsequently overlaid by GelBond film (80112933; Cytiva) cut to a circle, 12 mm in diameter, with the hydrophobic side facing down. The 12-well plate was placed in an inverted position for 40 min while the gel polymerized. Following gel polymerization, 2 mL 10× PBS was added to each well for 40 min prior to removal of the GelBond support. Gels were then washed 2× for 4 min in 1× PBS and subsequently treated with 40 µl Sulfo-SANPAH (sulfosuccinimidyl 6-(4’-azido-2’-nitrophenylamino) hexanoate; 22589; Thermo Fischer Scientific) for 4 min under ultraviolet (UV) light. The UV-treated Sulfo-SANPAH was then replaced with fresh Sulfo-SANPAH and treated with UV light for another 6 min. Following Sulfo-SANPAH cross-linking, gels were washed 2× for 4 min in 1x PBS and subsequently overlaid with 0.1 mg/mL collagen IV (C7521; Merck) and placed at 4 °C overnight. The next day, gels were washed twice in 1× PBS and then stored at 4 °C in 1× PBS for a maximum of 2 d before use in TFM experiments.

### TFM and Monolayer Stress Microscopy.

The microscope used for TFM was a Zeiss AxioObserver.Z1 equipped with a CO_2_ incubation chamber, a Colibri 7 LED light source, automated stage controlled by the Zen software, and a 10× 0.5 NA FLUAR air objective. Cells were seeded on collagen-coated PAA gels (4.0 kPa) in 12-well plates at 600,000 cells per well. Following growth for 12 to 20 h in normal growth medium, cells were subjected to serum deprivation for 48 h prior to serum restimulation and image acquisition. Subsequently, cells were placed on the microscope stage and allowed to adapt to the microscope environment for 1 h. Then, three to four consecutive frames (16 min intervals between frames) were captured in order to acquire a before stimulation reference. Imaging was then paused while a final concentration of 15% FBS with or without an inhibitor was added in order to serum activate the cell sheets. For each imaging time point, a total of four sites per well were captured. The 10× objective generated a field of view of 1,331 × 1,331 µm, and for each site, a stack of six z-planes was collected with a z-step size of 2.2 µm. For each z-stack, the middle z-plane was focused at the gel surface. The time interval between frames was 16 min and two channels were captured, namely, one for the beads (580/605 nm) and one for transmitted light to capture cell movements. At the end of image acquisition, cells were subjected to trypsin treatment for 1 h in order to capture a final reference frame of unstrained fluorescent beads.

To calculate traction forces and intercellular stress, each of the z-stacks from individual sites was projected using a maximal intensity projection algorithm. Subsequently, all images in a time series were subjected to image registration using the descriptor-based series registration plugin in Fiji ImageJ (59). Bead displacement, traction forces, and tension maps were calculated using open source codes available from pyTFM (60). In brief, this module calculates traction forces from the bead displacement fields by use of unconstrained Fourier transform traction cytometry (61). For calculation of monolayer stress, pyTFM uses the algorithm developed by Tambe et al. (62). Traction forces are given in Pascal (Pa), while the intercellular stress is given as the absolute value of the stress distribution (|σ|) as mN/m in all figures.

### Kymographs.

Spatiotemporal diagrams (kymographs) were created from time lapse images by particle image velocimetry (PIV) or TFM maps using an in-house Python-based script. In brief, one-dimensional images were generated by averaging pixel values along the *y* axis. Two-dimensional (2D) kymographs were then created by stacking the one-dimensional images.

### Monolayer Thickness Measurements.

HaCaT LifeAct-RFP/EGFP-Histone H2B cells were seeded at confluent cell densities in 35-mm glass bottom dishes from MatTek (P35G-1.5-14-C; MatTek Corporation). Volumetric stacks of living cell monolayers were generated at various cell densities following serum depletion for 48 h and subsequent serum reactivation for 18 h. The microscope used was a Leica TCS SP8 confocal microscope equipped with a 40× oil immersion objective, an incubation chamber maintaining 37 °C and 5% CO_2_, Hybrid (HyD) detectors, and the LAS X software. For each field of view (291 × 291 µm) the average monolayer thickness was calculated by measuring the depth of the LifeAct-RFP signal at four randomly selected positions, while cell density in the same image was estimated based on EGFP-labeled Histone H2B using the Find Maxima function in Fiji ImageJ. By fitting a linear polynomial to the average thickness data, we obtained a function that describes the correlation between monolayer thickness and cell sheet density. This function was used to calculate the cell sheet thickness in high-content imaging experiments with 96-well plates, based on monolayer density measurements acquired with the Find Maxima function.

### Visualization of Basal Actin Dynamics.

HaCaT LifeAct-RFP/EGFP-Histone H2B cells were seeded at confluent cell densities in 35-mm glass bottom dishes from MatTek (P35G-1.5-14-C; MatTek Corporation). Cells were subjected to 48 h of serum depletion followed by serum restimulation. Imaging of the basal cell surface after stimulation was carried out using a Leica TCS SP8 confocal microscope equipped with a 40× oil immersion objective and an incubation chamber maintaining 37 °C and 5% CO_2_. A time lapse series of a single Z-position representing the basal cell surface was acquired at a frame rate of 30 s between frames.

### Neighbor Cell Exchange Analysis.

For visualization and inspection of neighbor cell exchange in collectively migrating HaCaT cell sheets, we used a Fiji ImageJ macro that performs image-based registration using a single cell as a tie point (63). In brief, for each dataset, a randomly selected cell was marked as a point, creating a set of regions of interest (ROIs) through the time series. Subsequently, images (200 x 200 pixels) representing a pack of 20 to 40 cells were automatically generated keeping the specific ROI in the center of each image. This creates time lapse videos, where one selected cell is in the center of each frame and potential neighbor cell exchange can be studied.

### Cell Division Analysis.

HaCaT mCherry-Histone H2B cells were seeded in 12-well glass bottom multiwell plates from MatTek (P12G-1.5-14-F; MatTek Corporation) at 600,000 cells per well. Notably, all cells were seeded 12 to 20 h before the start of serum depletion. Cells were subjected to different time periods of starvation, followed by serum restimulation and widefield microscopy. Image acquisition was carried out on a Zeiss AxioObserver.Z1 microscope using a 10× 0.5 NA FLUAR air objective, a filter set for the detection of mCherry fluorescence, and a CO_2_ incubation chamber. Four randomly selected sites per well were acquired for a total time period of 50 h, using a time interval of 16 min between frames. First, mitotic cells in a set of images were annotated and used as input data for development of a deep learning model using the StarDist (2D) network, available in the CoLab notebook (64). Second, the deep learning model was further used to detect cell division frequencies in each frame of complete datasets, by running the model in the StarDist plugin of Fiji ImageJ (65).

### Mathematical Model.

A minimal mathematical model for the epithelial layer, considered to behave as a continuum, is based on an active gel theory (45–51) with contributions from elastic deformations, active actinomyosin contractions, viscous friction, and cell–substrate friction. The monolayer elastic displacement field is u(x, t) with x the spatial coordinate vector and t time. The active actinomyosin is labeled by a concentration field c(x,t), (66–68). During cell migration, the traction force on the underlying substrate becomes

[1] $$\text{T}=\Gamma \partial_{t}u(x,\;t),$$

with Γ [Ns m^−3^] as the cell–substrate friction coefficient. The monolayer stress consists of a passive elastic stress $\sigma_{\mathrm{el}}(x,t)$, which is modeled as a linearly elastic material (69), and an active stress $\sigma_{\alpha }(x,t)$. We assume a linear relation between c(x,t) and the active stress (48). The dynamics of the monolayer is then given by the force balance

[2] $$\Gamma \partial_{t}u(x,t)=h_{eq}E\nabla \cdot \underline{\underline{\epsilon }}\left(x,t\right)+h_{eq}\eta \partial_{t}\nabla \cdot \underline{\underline{\epsilon }}\left(x,t\right)+h_{eq}\alpha \nabla c\left(x,t\right),\;$$

where E [Pa] is the Young’s modulus, $\underline{\underline{\epsilon }}=\frac{1}{2}(\nabla u\left(x,t\right)+\nabla u{\left(x,t\right)}^{T})$ is the strain tensor, $h_{eq}$ [m] is the average monolayer thickness, η is the cell sheet viscosity, and α is the contractile strength.$\;\partial_{t}$ is the partial derivative with respect to time and ∇ is the 2D gradient operator. c(x,t) is described by a convection-diffusion-reaction equation

[3] $$\partial_{t}c\left(x,t\right)+\partial_{t}u\left(x,t\right)\cdot \nabla c\left(x,t\right)=-\frac{1}{\tau_{c}}\left(c\left(x,t\right)-c_{eq}\right)+\beta \nabla \cdot \underline{\underline{\epsilon }}\left(x,t\right)+D\nabla^{2}c\left(x,t\right),\;$$

where $\tau_{c}$ [s] is the relaxation time scale toward the equilibrium concentration $c_{eq}$ [-] that is defined as the concentration at which there are no intracellular contractions, β [t^−1^] is the activation rate due to stretching or compression in the monolayer, and D [m^2^ s^−1^] is a diffusion coefficient. Since η primarily affects the time scale of the dynamics and not the qualitative behavior of the cell sheet displacements, only results for η=0 are shown in the main text. The influence of η and a further discussion of the model, the choice of the material parameters, and their sensitivity is discussed in *SI Appendix*.

### Numerical Simulations.

The mathematical model represented by Eqs. 2 and 3 is solved using a Newton solver from the finite element library FEniCS (70), where the spatial gradients are discretized by a piecewise linear function. The 2D circular mesh with non-dimensional radius equal to one isgenerated with the software Gmsh (71) using a mesh spacing of 0.015. The boundary conditions used in the simulations are as follows: no displacement u(|x|=1,t)=0 and a no flux of c(x,t), ∇c(x,t)·n=0. We seed 10 meshes with an initial condition for c(x,t=0), where each spatial mesh point have a 60% chance of being seeded with a random number between 0 and 1 drawn from a Gaussian distribution. For each initial seeding, we simulate the monolayer dynamics by multiplying the concentration with a prefactor, here [0.01, 0.1, 0.5, 1.0, 2.0, 3.0] giving different values for $c_{0}$, before solving **[2]** to **[3]**. We use an initial nondimensional time step of $5\cdot 10^{-5}\;$ that is increased by 5% for each time step until a maximum time step of 10^−3^ is reached that is then kept constant until a nondimensional time 3 at which the simulations are terminated. This is sufficiently long for all simulations that display the formation of one large central contraction center that has reached its maximum contraction. When the simulations are allowed to further continue in time, the layer eventually flattens with a uniform $c(x,t)\;=\;c_{eq}$ field.

### Averaged Radial Velocity.

The radial velocity in the monolayer is obtained by calculating the radial displacement at each spatial point from the Cartesian displacement field u(x,t). The average velocity is then calculated by averaging over the 10 simulations for each prefactor to the initial concentration seeding with the SD being the maximum and minimum values obtained in the 10. Initially, the radial displacement field is random in the sense that it has approximately equal amounts of cells moving toward and away from the center due to the random concentration seeding and the subsequent formation of small local contraction centers. Therefore, we define the averaged radial velocity to be the averaged radial velocity values obtained in the time starting when less than 1% of the cells are moving away from the center to the time when the velocity is reduced to 20% of its initial magnitude.

## Supplementary Material

Supplementary File

Supplementary File

Supplementary File

Supplementary File

Supplementary File

Supplementary File

Supplementary File

Supplementary File

Supplementary File

## Data Availability

All study data are included in the article and/or supporting information.
