# Supplementary material for: Mechanical coupling of supracellular stress amplification and tissue fluidization during exit from quiescence
Source: Proc Natl Acad Sci U S A. 2022 Aug 1;119(32):e2201328119. doi: 10.1073/pnas.2201328119 (PMC9371707; doi:10.1073/pnas.2201328119)
Supplement: Supplementary File [file pnas.2201328119.sapp.pdf]

Supplementary Information for

Mechanical coupling of supracellular stress amplification and tissue fluidization during exit from quiescence

Emma Lång[1], Christian Pedersen[2], Anna Lång[1], Pernille Blicher[3], Arne Klungland[1,4], Andreas Carlson[2 *], and Stig Ove Bøe[1*]

[1]Department of Microbiology, Oslo University Hospital, 0373 Oslo, Norway;
[2]Department of Mathematics, Mechanics Division, University of Oslo, 0851 Oslo, Norway;
[3]Department of Medical Biochemistry, Institute of Clinical Medicine, University of Oslo, 0372 Oslo, Norway;
[4]Department of Biosciences, University of Oslo, 0371 Oslo, Norway.

[*]Correspondence:
Stig Ove Bøe
E-mail: stig.ove.boe@rr-research.no
Andreas Carlson
E-mail: acarlson@math.uio.no

This PDF file includes:

Supplementary methods
Figures S1 to S14
Tables S1 to S2
Legends for Movies S1 to S9
Supplementary References

Other supplementary materials for this manuscript include the following:

Movies S1 to S9

Supplementary Methods

Mathematical model

Here we give a detailed description of the model presented in the main text. We assume that the cellular monolayer is a circular layer of radius R and average thickness h_{eq}. In previous models of epithelial monolayers both cell based vertex or Voronoi models (1-4) and continuum models (1, 5) have been considered for which its constitutive equations described the monolayer as an elastic (6-9) or a viscous (10-12) material. Here, we assume that the monolayer can be described as a two-dimensional active gel (13, 14) with a displacement field $\mathbf{u}(\mathbf{x}, t)$ with $\mathbf{x}$ being the spatial coordinate, the bold style indicates vector notation and t is time. The displacement at a specific position is coupled to an activation concentration $c(\mathbf{x}, t)$, which regulates the contractile stresses within the cellular monolayer *e.g.* actinomyosin, which we adopt into the model to model the experimental measured behavior. Additional contributions to the model such as polarization of the cells are discussed in a following section.

With negligible inertial forces and assuming no shear stress on the monolayer free surface, the in-plane force balance averaged over the two-dimensional monolayer's height is given as

$$\mathbf{T}(\mathbf{x}, t) = h_{eq} \nabla \cdot \underline{\underline{\sigma}}(\mathbf{x}, t) \qquad \textbf{[I]}$$

where $\mathbf{T}(\mathbf{x}, t)$ is the traction forces generated by the friction between the monolayer and the substrate and $\underline{\underline{\sigma}}(\mathbf{x}, t)$ is the cellular stress tensor. The traction force is given as $\mathbf{T}(\mathbf{x}, t) = \Gamma \partial_t \mathbf{u}(\mathbf{x}, t)$ with Γ being the cell-substrate friction coefficient. ∂_t is the partial derivative with respect to time and ∇ is the two-dimensional gradient operator. The cellular stress consists of the sum of the passive elastic stress and the active stress due to the activation concentration (15) $\underline{\underline{\sigma}}(\mathbf{x}, t) = \underline{\underline{\sigma_p}}(\mathbf{x}, t) + \underline{\underline{\sigma_a}}(\mathbf{x}, t)$. The stress is modeled as a sum of a linearly elastic material $\underline{\underline{\sigma}}_{el} = E \underline{\underline{\epsilon}}$, where E is the Young's modulus, $\underline{\underline{\epsilon}} = 1/2 \left(\nabla \mathbf{u}(\mathbf{x}, t) + \left(\nabla \mathbf{u}(\mathbf{x}, t) \right)^T \right)$ is the strain tensor and a viscous component $\underline{\underline{\sigma}}_v(\mathbf{x}, t) = \eta \, \partial_t \underline{\underline{\epsilon}}(\mathbf{x}, t)$ with η the cellular viscosity. The active contribution is modeled with a linear dependence to the amount of active contractile units mimicked by the concentration field $c(\mathbf{x}, t)$ giving $\underline{\underline{\sigma_a}}(\mathbf{x}, t) = \alpha c(\mathbf{x}, t) \underline{\underline{I}}$, where α sets the magnitude of the isotropic contractile force and $\underline{\underline{I}}$ is the identity matrix. The dynamics of the cellular monolayer is described by the force balance between the intracellular stresses generated by the amount of activated actinomyosin $(c(\mathbf{x}, t))$ and the traction forces acting on the monolayer from the underlying substrate. Considering the monolayer to be an active gel, the force balance in the continuum limit is expressed as

$$\Gamma \, \partial_t \mathbf{u}(\mathbf{x}, t) = h_{eq} E \nabla \cdot \underline{\underline{\epsilon}}(\mathbf{x}, t) + h_{eq} \eta \, \partial_t \nabla \cdot \underline{\underline{\epsilon}}(\mathbf{x}, t) + \alpha \nabla c(\mathbf{x}, t). \qquad \textbf{[II]}$$

The dynamics of the concentration field is given as a convection-reaction-diffusion equation

$$\partial_t c(\mathbf{x}, t) + (\partial_t \mathbf{u}(\mathbf{x}, t) \cdot \nabla) c(\mathbf{x}, t) = \frac{-1}{\tau_c} \left(c(\mathbf{x}, t) - c_{eq} \right) + \beta \nabla \cdot \mathbf{u}(\mathbf{x}, t) + D \nabla^2 c(\mathbf{x}, t) \qquad \textbf{[III]}$$

τ_c is the relaxation time scale towards an equilibrium concentration c_{eq} that eventually will lead to a flat homogenous stationary monolayer, β is the rate of production of concentration due to cellular contraction and D is the diffusion coefficient. The terms on the left-hand side of equation **[III]** describes the rate change and convection of $c(\mathbf{x}, t)$, respectively. On the right-hand side, the first term gives the kinetic growth/decay around an equilibrium concentration. The second term represents the increase or decay in activation during stretching/compression of the cells and the last term describes the diffusion of the signal inside the layer.

Biophysical parameters

All model parameters are cell-type dependent and are chosen accordingly when rescaling the data obtained from the numerical simulations. The monolayer thickness, h_{eq}, the monolayer radius, R, and the Young's modulus of the monolayer, E, are measured experimentally and are listed in Supplementary Table 1. The remaining values are chosen within the range reported in the literature (6, 9), and their exact value are determined by comparing our numerical results with the experimentally measured time and length scales when mapping out the phase space in cell layer dynamics.

Supplementary Table 1: Model parameters.

Parameter	Description	Reference value	Measured value	Values in main text
Γ	Cell-substrate friction coefficient	10^7-10^9 Ns m^{-3} (9, 16)	-	$2.5 \cdot 10^8$ Ns m^{-3}
E	Monolayer Young's modulus	-	4000 Pa	4000 Pa
α	Coupling coefficient between displacement and concentration	200 Pa (9)	-	100 Pa
τ_c	Concentration relaxation time scale	4200 – 21000 s (9, 16)	-	13000 s
β / c_{eq}	Production rate of concentration due cellular compression	$3 \cdot 10^{-5}$ – $6 \cdot 10^{-4}$ s^{-1} (9, 16)	-	$8 \cdot 10^{-5}$ s^{-1}
D	Diffusion coefficient	-	-	10^{-9} $m^2 s^{-1}$
h_{eq}	Average monolayer thickness at equilibrium	-	8 µm	8 µm
R	Monolayer radius	-	3.6 mm	3.6 mm
f	Strength of polarization-displacement coupling	10 Pa (9)	-	10 Pa
a	Polarization relaxation rate	$2.16 \cdot 10^{-4}$ s^{-1} (9)	-	$2.16 \cdot 10^{-4}$ s^{-1}
κ	Nearest neighbor alignment for **p**	$1.46 \cdot 10^{-13}$ $m^2 s^{-1}$ (9)	-	$1.46 \cdot 10^{-13}$ $m^2 s^{-1}$
ω	Polarization alignment coefficient with the gradient of c	3.47 ms^{-1} (9)	-	3.47 ms^{-1}
η	Tissue viscosity	10-10^7 Pa s (11, 12, 17)	-	0 Pa s

Velocity scaling

To gain some insight into the model predictions and what effect the active concentration have on the monolayer displacement we turn to a scaling analysis of equation **[II]**. We assume the gradient ∇ to scale as L^{-1}, with L being a characteristic length scale of the system and $\partial_t \sim t^{-1}$ and scale $c(\mathbf{x},t) \sim c_0$. By using these scaling arguments we can deduce the cellular displacement scales as

$$u\left(\frac{\Gamma}{t} - \frac{h_{eq}E}{L^2} - \frac{h_{eq}\eta}{L^2 t}\right) \sim \frac{h_{eq}\alpha}{L} c. \qquad \textbf{[IV]}$$

The velocity in the monolayer, $\sim u/t$, can then be estimated as

$$\frac{u}{t}\left(1 - \frac{h_{eq}E}{\Gamma L^2} t - \frac{h_{eq}\eta}{\Gamma L^2}\right) \sim \frac{h_{eq}\alpha}{\Gamma L} c. \qquad \textbf{[V]}$$

At early times and small displacement we see that the monolayer velocity scales linearly with the concentration gradient, as is recovered with a small offset in the numerical simulations and shown in Supplementary Figure 5, where the maximal velocity is plotted as a function of the initial concentration ratio c_0. Moreover, as the monolayer contracts the concentration in the compressed regions will be deactivated and thus reducing the monolayer velocity. From equation **[V]** we see that the time scale for the velocity is $(h_{eq}E/(\Gamma L^2))^{-1}$, which for the values listed in Supplementary Table 1 yields $t \approx$ 28h in correspondence with the time scale observed in the experiments when using the monolayer radius R as the characteristic length.

Numerical simulations

We non-dimensionalize equations **[II]** - **[III]** by introducing the scaling parameters $\mathbf{x} = \hat{\mathbf{x}}R$, $t = \hat{t}\,\Gamma R^2/(Eh_{eq})$, $\mathbf{u} = \hat{\mathbf{u}}R$ and $c = \hat{c}c_{eq}$ with the hat notation indicating non-dimensional variables. The non-dimensional version of equations **[II]** - **[III]** then becomes

$$\partial_{\hat{t}}\hat{\mathbf{u}}(\hat{x},\hat{t}) = \hat{\nabla}\cdot\underline{\underline{\epsilon}}(\hat{x},\hat{t}) + \hat{\eta}\,\partial_{\hat{t}}\hat{\nabla}\cdot\underline{\underline{\epsilon}}(\hat{x},\hat{t}) + \hat{\alpha}\hat{\nabla}\hat{c}(\hat{x},\hat{t}) \qquad \textbf{[VI]}$$

$$\hat{\Gamma}_c\left(\partial_{\hat{t}}\hat{c}(\hat{x},\hat{t}) + \partial_{\hat{t}}\hat{\mathbf{u}}(\hat{x},\hat{t})\cdot\hat{\nabla}\hat{c}(\hat{x},\hat{t})\right) = -\hat{\tau}_c(\hat{c}(\hat{x},\hat{t}) - 1) + \hat{\beta}\hat{\nabla}\cdot\hat{\mathbf{u}}(\hat{x},\hat{t}) + \hat{\nabla}^2\hat{c}(\hat{x},\hat{t}) \qquad \textbf{[VII]}$$

with the dimensionless parameters $\hat{\eta} = \eta h_{eq}/(\Gamma L^2)$ which is the ratio of viscous damping over friction forces, $\hat{\alpha} = \alpha c_{eq}/E$ being the ratio between the active contractile strength and the elastic stiffness, $\hat{\Gamma}_c = Eh_{eq}/(D\Gamma_u c_{eq})$ controls the strength between the elastic response and the friction due to diffusion of concentration, $\hat{\tau}_c = R^2/(D\tau_c)$ is the kinetic time scale towards an equilibrium concentration, and $\hat{\beta} = \beta R^2/(Dc_{eq})$ is the ratio between the deactivation of concentration due to the monolayer compression and concentration diffusion. We solve the set of equations **[VI]** – **[VII]** coupled using an implicit Newton solver from the finite element library FEniCS on a circular mesh. The numerical simulations are initiated with a zero displacement condition but with a randomized concentration to mimic the stress that is generated in the monolayer during quiescence. The randomization of the initial concentration is performed such that each spatial coordinate has a 60% chance of being seeded with a value drawn from a Gaussian distribution. This initial concentration is then multiplied with a pre-factor that corresponds to the starvation period in the experiments, *i.e.* large pre-factor equals long

starvation period. The initial concentration is defined as $c_0 = \sum \hat{c}(\hat{\mathbf{x}},\ \hat{t} = 0)\ dA\ /\ c_{eq}$, with dA the area of the triangulated mesh. We perform 10 simulations from which we compute the average of the system variables. The boundary conditions are set to reflect what is observed experimentally and we therefore use a homogenous Dirichlet condition on the displacement field $\hat{\mathbf{u}}(\hat{\mathbf{x}} = \partial\Omega,\ \hat{t}) = 0$ and a homogenous Neumann condition on the concentration, $\hat{\nabla}\hat{c}(\hat{\mathbf{x}} = \partial\Omega,\ \hat{t}) \cdot \mathbf{n} = 0$ with $\mathbf{n}$ being the normal vector to the domain boundary $\partial\Omega$. We multiply equations **[VI]** - **[VII]** with the test functions $\phi(\hat{x})$, $\psi(\hat{x})$ and integrate by parts over our numerical domain Ω to obtain the variational formulation of our system equations as

$$\int_\Omega \partial_{\hat{t}}\hat{\mathbf{u}} \cdot \phi d\hat{x} + \int_\Omega (1 + \eta\, \partial_{\hat{t}})\underline{\underline{\hat{\epsilon}}} \cdot \hat{\nabla}\phi d\hat{x} - \int_{\partial\Omega} (1 + \eta\, \partial_{\hat{t}})\underline{\underline{\hat{\epsilon}}} \cdot n\phi ds - \int_\Omega \hat{\alpha}\hat{\nabla}\hat{c} \cdot \phi d\hat{x} = 0, \quad \textbf{[VIII]}$$

$$\int_\Omega \hat{\Gamma}_c\left(\partial_{\hat{t}}\hat{c} + \partial_{\hat{t}}\hat{\mathbf{u}} \cdot \hat{\nabla}\hat{c}\right)\psi d\hat{\mathbf{x}} + \int_\Omega \hat{\tau}_c(\hat{c} - 1)\psi d\hat{\mathbf{x}} - \int_\Omega \hat{\beta}\hat{\nabla} \cdot\ \hat{\mathbf{u}}\psi d\hat{\mathbf{x}} + \int_\Omega \hat{\nabla}\hat{c} \cdot \hat{\nabla}\psi d\hat{\mathbf{x}} = 0. \quad \textbf{[VIIII]}$$

As our equations are reduced to first order equations, we discretize them using linear elements with the implicit discrete time derivatives as

$$\frac{\hat{\mathbf{u}}^n - \hat{\mathbf{u}}^{n-1}}{\Delta\hat{t}} = f(\hat{\mathbf{u}}^n, \hat{c}^n) \quad \textbf{[X]}$$

$$\frac{\hat{c}^n - \hat{c}^{n-1}}{\Delta\hat{t}} = g(\hat{\mathbf{u}}^n, \hat{c}^n) \quad \textbf{[XI]}$$

with n being the current time step we evaluate, $\Delta\hat{t}$is the time step spacing and f, g represents the remaining terms in equations **[VIII]** – **[VIIII]**, respectively.

Supplementary Table 2*:* Non-dimensional model parameters.

Parameter	Description	Tested value range	Values in main text
$\hat{\alpha}$	Ratio between contractile strength and elastic stiffness	0.01 – 10	10
$\hat{\beta}$	Deactivation of concentration due to monolayer compression	$10^{-2} – 10^{4}$	1
$\hat{\Gamma}_c$	Strength between elastic response and substrate friction	$10^{-4} – 10^{2}$	100
$\hat{\tau}_c$	Concentration relaxation time scale	$10^{-2} – 10^{2}$	1
$\hat{f}$	Displacement-polarization coupling coefficient	1.125	1.125
$\hat{a}$	Polarization relaxation time scale	22	2
$\hat{\kappa}$	Strength of nearest neighbor alignment	10^{-3}	10^{-3}
$\hat{\omega}$	Polarization alignment rate with concentration gradient	0.975	0.975
$\hat{\eta}$	Ratio between viscous and friction forces	0-1	0

Parameter sensitivity – contraction center formation

To determine the effect from the non-dimensional system parameters on the dynamics we performed a parameter sensitivity study, where we systematically vary two parameters while

keeping all others fixed. All simulations are initialized with the same initial condition depicted in Supplementary Figure 7 with a ratio $c_0 = 1.2$.

The results from the study are shown in Supplementary Figures 8, 9, 10, 13 with the red dotted lines highlighting the parameter range that yields collective motion and formation of a contraction center, and is summarized in the following points:

- $\hat{\alpha}$ determines the magnitude of the monolayer displacement for a given value of c_0. A sufficiently large value of $\hat{\alpha}$ is crucial to obtain the large-scale migration needed to form one big central contraction center.

- The dynamics are unaffected by small values of $\hat{\beta}$ yet it can be important with large values of $\hat{\alpha}$ to control the maximum central compression. For large values of $\hat{\beta}$ the concentration deactivation due to the monolayer compression can stop the collective migration and prevent the formation of a central contraction center.

- Smaller values of $\hat{\Gamma}_c$ lead to smoother and more symmetric migration due to diffusion of the active contractile units yet with a reduced displacement field magnitude. Larger values of $\hat{\Gamma}_c$ increase the displacement magnitude but not to the same extent as $\hat{\alpha}$. A sufficiently large value of $\hat{\Gamma}_c$ is needed to obtain the convective dynamics observed in the experiments.

- Large values of $\hat{\tau}_c$ increase the deactivation rate of the concentration to such an extent that collective migration can be halted but very large values are needed to significantly affect the dynamics.

- Increasing values of $\hat{\eta}$ changes the time scale of the cellular contraction. We also see that the magnitude of the contraction centers is fairly insensitive to the value of $\hat{\eta}$.

As $\hat{\eta}$ does not affect the characteristics of the large-scale contraction as shown in Supplementary Figure 13 we neglect the viscous contribution in the following where the viscous term can also be absorbed into the uncertainty associated with the cell-substrate friction coefficient. Moreover, from our non-dimensional number $\hat{\eta} = \eta h_{eq}/(\Gamma L^2)$ we see, using the parameter values in Supplementary Table 1, that obtaining a value of $\hat{\eta} = 1$ requires in this model a fairly large cellular viscosity. A much smaller value of $\hat{\eta}$ is likely relevant in our system that would not significantly affect the dynamical behavior. Although the viscosity does not appear to influence the dynamics of the active contractile motion, it can be predominant in the long time relaxation of the monolayer or in the presence of cell division and apoptosis, which has been observed in other studies (17, 18).

To extend our analysis in the absence of viscous forces we plotted the maximum monolayer velocity magnitude, $\max(|\partial_{\hat{t}}\hat{\mathbf{u}}|)$, from the simulation data that provided the displacement fields in Supplementary Figures 8-10, shown in Supplementary Figure 11. The previously summarized conclusions are further enhanced and we see that the velocity magnitude is approximately unaffected by changes in both $\hat{\tau}_c$ and $\hat{\beta}$. Moreover, we see that the monolayer velocity magnitude increases linearly with $\hat{\alpha}$ and the square root of $\hat{\Gamma}_c$, making them both important to

achieve large-scale collective migration.

We can thus separate the four dimensionless parameters into two categories, with $\hat{\alpha}$ and $\hat{\Gamma}_c$ being the parameters important for initiating the collective migration, and $\hat{\tau}_c$ and $\hat{\beta}$ are important to slow down the migration at late times, preventing possible diverging compression in the monolayer and to enforce the inevitable relaxation to a flat homogenous equilibrium monolayer at $\hat{t} \rightarrow \infty$.

Numerical solution in a square geometry

In order to check if the collective migration towards the center of the circular mesh is a feature induced by the geometry, we solve the same system on a square mesh. In Supplementary Figure 12A the displacement field in the square geometry shows that the formation of a large central contraction center occurs also for non-circular meshes. Moreover, the displacement field magnitude only differs by ~ 10% to that in the circular mesh (Supplementary Figure 12B) given the same non-dimensional parameters $\hat{\alpha} = \hat{\beta} = \hat{\tau}_c = \hat{\Gamma}_c = 1$.

Model and experiments - comparison

We performed numerical simulations using a wide range of initial concentration ratios and verified that an initial concentration can cause a collective cellular migration behavior towards the center of the monolayer. The initial response to the random concentration is to form small local contraction centers of high cell density, where the peaks in the initial concentration are located, accompanied by a large traction force pointing in towards these centers. After these initial density centers were formed the monolayer displacement was directed inwards toward the center of the monolayer, where one large cell density center was formed. Below a threshold ratio value c_0, there was no collective motion towards the center but the monolayer relaxed to equilibrium after the initial formation of the small density centers (Supplementary Video 7). Around the threshold ratio value stronger local density centers were formed with a subsequent collective motion towards the center of the mesh. However, with small gradients in the concentration the dynamics stagnated before a single central contraction center was formed (Supplementary Video 9). Above the threshold value a grand collective migration occurred towards the center of the mesh, after the initial formation of small local contraction centers. At these conditions the dynamics did not stagnate until a high density central contraction center was formed (Supplementary Video 8). Thus, the dynamics produced in our numerical simulations using a sufficiently high value of the initial concentration ratio c_0 recapitulates the dynamic behavior observed in serum-stimulated quiescent cell monolayers (Figure 1, Supplementary Video 2). The traction force was shown to peak at the early formation of the small local contraction centers and decay as the cells began to migrate collectively, which is in agreement with the experimental observations (see Figure 2A and F). The results from the numerical simulations are shown in Figure 4J. These data also highlights that the traction force magnitude increases with the initial concentration ratio c_0. The overall traction force profile is qualitatively similar to that measured in the experiments, shown in Figure 2F-G. In addition to the increased traction force magnitude, a larger value of c_0 also indicated an increased migration velocity. The averaged radial migration velocity calculated from the numerical data is shown in Figure 4K.

The data from the numerical simulations exhibit an increase in the radial velocity for initial

concentrations (c_0) larger than 1, followed by saturation in migration speed for values above 1.3. From the numerical data we could fit a sigmoid function that describes the migration velocity as a function of initial concentration (Figure 4K). To bridge the gap between the initial concentration (c_0) in the model and the starvation period in the experiments we plotted the extracted sigmoid function together with the experimental observations representing cell migration velocity versus quiescence depth (starvation period) in Figure 5B. This demonstrates a good agreement between the experimental results and the phenomenological model.
We also perturbed our system to mimic the monolayer dynamics in the presence of actinomyosin inhibitors. To achieve this, we reduced the value of the non-dimensional parameter $\hat{\alpha}$, which controls the contractile strength in the model. During the parameter study (see Supplementary Figure 8 and Figure 4G-I) we show that our model exhibits no large-scale migration for small values of $\hat{\alpha}$, and in Supplementary Figure 4 we demonstrate that reducing the value of $\hat{\alpha}$ leads to decreased traction force magnitude, which is consistent with the experimental observations presented in (Figure 3A and B and Figure 5A).

Polarization effects
From the experiments there is no indication that the collective motion and formation of a contraction center is driven by cellular polarization. However, we know that cellular polarization can have an effect on the monolayer dynamics (3, 6-9, 12, 19).
To investigate the effects that cellular polarization has on the dynamics we introduced a polarization vector $\mathbf{p}(\mathbf{x}, t)$. Following (9), the dynamics of the polarization field can be described by the following equation

$$\partial_t \mathbf{p}(\mathbf{x},\mathrm{t}) = a(1 - |\mathbf{p}^2(\mathbf{x},\mathrm{t})|)\mathbf{p}(\mathbf{x},\mathrm{t}) + \kappa\nabla^2\mathbf{p}(\mathbf{x},\mathrm{t}) + \omega\nabla c(\mathbf{x},\mathrm{t}) \qquad \textbf{[XII]}$$

with a controlling the rate of relaxation towards a homogeneously polarized monolayer, κ is the strength of the nearest neighbor alignment, and ω is the coupling coefficient to the concentration gradient, such that local cell polarization points towards regions of high concentration. Further, we coupled the polarization field to the monolayer displacement through the traction force, which now reads $\mathbf{T}(\mathbf{x}, t) = \Gamma\partial_t\mathbf{u}(\mathbf{x}, t) - f\mathbf{p}(\mathbf{x}, t)$, with f being the coupling coefficient between the cell polarization and the monolayer displacement. Inserted into equation **[I]** we get

$$\Gamma\partial_t \mathbf{u}(\mathbf{x},\mathrm{t}) = h_{eq}E\nabla\cdot\underline{\underline{\epsilon}}(\mathbf{x},\mathrm{t}) + h_{eq}\alpha\nabla c(\mathbf{x},\mathrm{t}) + f\mathbf{p}(\mathbf{x},\mathrm{t}). \qquad \textbf{[XIII]}$$

We non-dimensionalize equations **[XII]** - **[XIII]** as in the Numerical methods section to obtain

$$\partial_{\hat{t}}\hat{\mathbf{u}}(\hat{x},\hat{t}) = \nabla\cdot\underline{\underline{\epsilon}}(\hat{x},\hat{t}) + \hat{\alpha}\hat{\nabla}\hat{c}(\hat{x},\hat{t}) + \hat{f}\hat{\mathbf{p}}(\hat{x},\hat{t}) \qquad \textbf{[XIV]}$$

and

$$\partial_{\hat{t}}\hat{\mathbf{p}}(\hat{x},\hat{t}) = \hat{a}(1 - |\hat{p}(\hat{x},\hat{t})|^2)\hat{p}(\hat{x},\hat{t}) + \hat{\kappa}\hat{\nabla}^2\hat{\mathbf{p}}(\hat{x},\hat{t}) + \hat{\omega}\hat{\nabla}\hat{c}(\hat{x},\hat{t}) \qquad \textbf{[XV]}$$

with $\hat{f} = fR/(h_{eq}E)$ being the strength of the polarization-displacement coupling, $\hat{a} = a\tau$ the polarization relaxation rate, $\hat{\kappa} = \kappa\tau/R^2$ the local neighbor alignment coefficient, and $\hat{\omega} = \omega\tau c_{eq}/R$ is the strength of the polarization alignment with the concentration gradient. We now have a non-dimensional coupled three equation system consisting of equations **[XIV]**, **[VII]** and **[XV]** that we solve as described in the Numerical methods section. Using parameter values found in the literature, see Supplementary Table 1 (Supplementary Table 2 for non-dimensional units), we see the results from a numerical simulation in Supplementary Figure 14. At three different times we compared the displacement field with a polarization field to the displacement field without a polarization field using the same initial condition displayed in Supplementary Figure 7. There were no significant differences in displacement magnitude, length scale or time scale between polarized and unpolarized results. This indicates that cellular polarization has no significant impact on the large-scale collective migration observed in this study, which is consistent with the experiments and we use this as a justification to neglect this effect in our theoretical model.

Supplementary Figures

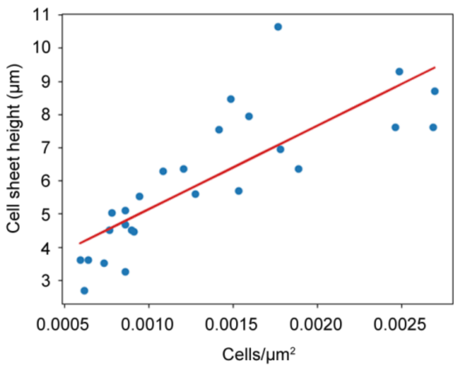

Supplementary Figure 1. Monolayer thickness measurements. Manual measurements of cell sheet thickness were performed on confluent keratinocyte monolayers subjected to serum depletion for two days followed by serum re-stimulation. Graph shows average values (blue dots) of cell sheet thickness at different cell densities. The red line shows the best fit curve based on linear regression.

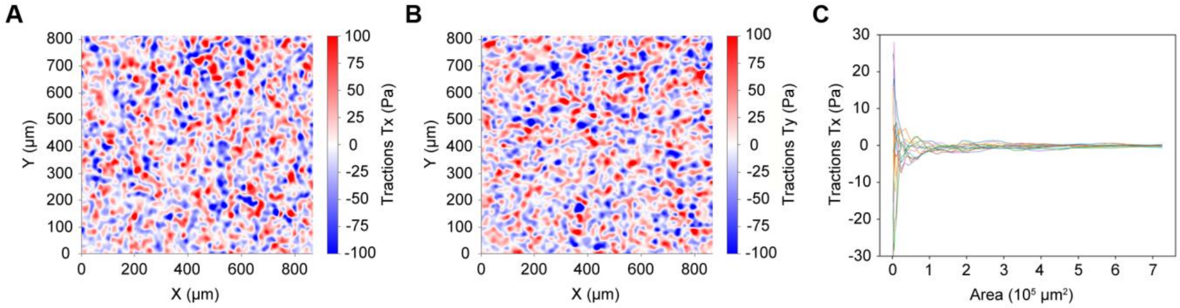

Supplementary Figure 2. Random orientation of local traction forces. (A-B) Plotting of individual traction force components T_x (A) and T_y (B) across a microscopic field of view (866x814 µm). (C)

Plot showing the average of the traction force component T_x within a progressively increasing microscopic field area. The time point selected is 1 h after serum stimulation.

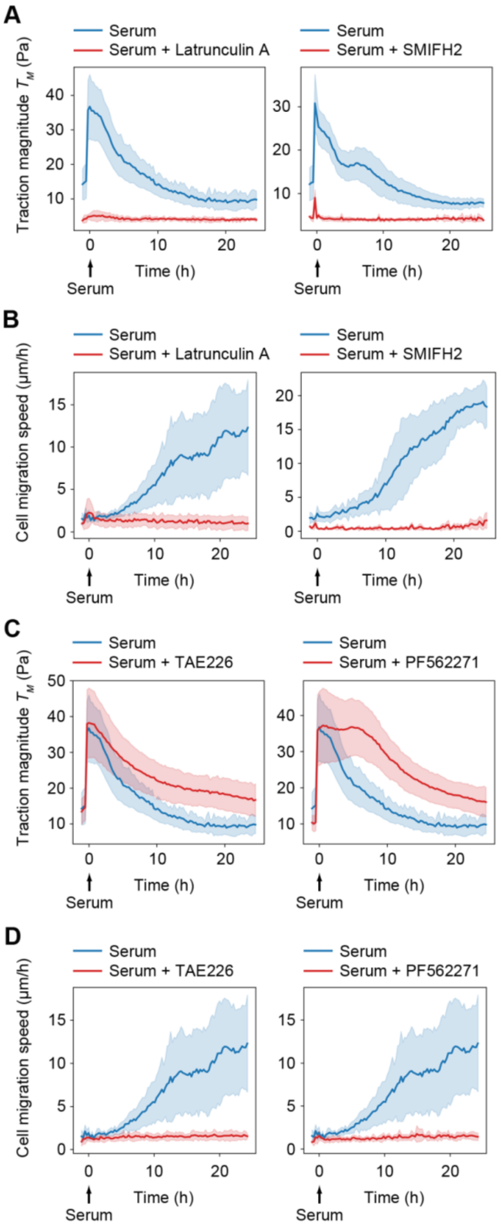

Supplementary Figure 3. Dependency between global stress amplification and cell sheet displacement. (A) Traction force magnitudes and (B) cell migration speed during serum activation in the presence of the actinomyosin inhibitors Latrunculin A or SMIFH2. (C) Traction force magnitudes and (D) cell migration speed generated in the presence of collective cell migration inhibitors specific for FAK (TAE226 and PF562271). (A-D) The time of serum stimulation (t=0) is indicated. All graphs show average values ± SD from 8 separate microscopy fields.

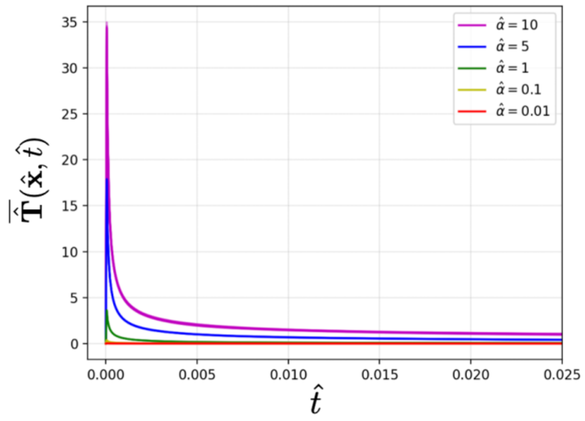

Supplementary Figure 4. Arithmetic mean of the non-dimensional traction force magnitude $\overline{\hat{T}}(\hat{x}, \hat{t})$ for different values of $\hat{\alpha}$. The remaining parameters values are set to that indicated in Supplementary Table 2.

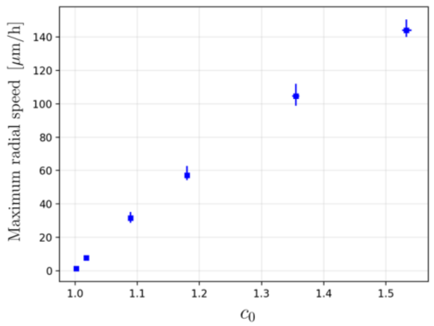

Supplementary Figure 5. Maximum radial velocity in the monolayer as calculated from the numerical simulations. The maximum velocity follows a linear trend with the initial concentration magnitude with a small decaying offset.

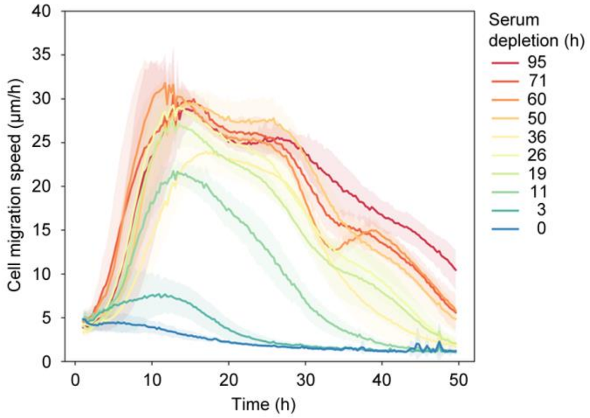

Supplementary Figure 6. Line plots showing changes in cell sheet migration speed over time after different time periods of serum depletion prior to serum re-activation of confluent cell sheets. Graph shows mean values ± SD.

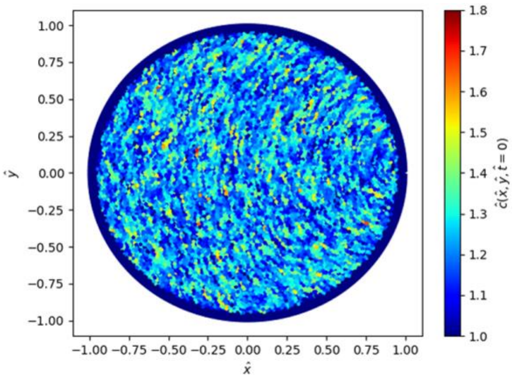

Supplementary Figure 7. Initial condition for the concentration $\hat{c}(\hat{\mathbf{x}},\ \hat{t}\ =\ 0)$ for all the simulations in the parameter study. The initial condition has a ratio c_0 = 1.2.

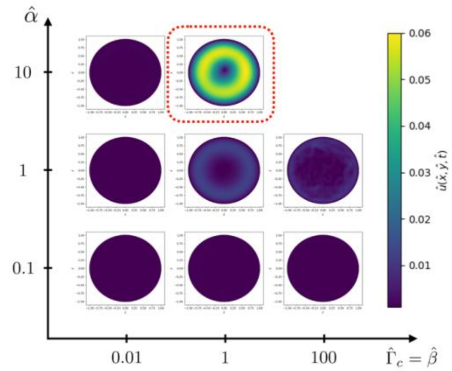

Supplementary Figure 8. Displacement field $\hat{\mathbf{u}}(\hat{\mathbf{x}},\ \hat{t})$ at the time of maximal contraction plotted as a function of $\hat{\Gamma}_c$ and $\hat{\tau}_c$. In all the simulations $\hat{\tau}_c = 1, \hat{\eta} = 0$ and $c_0 = 1.2$. The red dashed line marks the parameter space for which a global contraction center is formed.

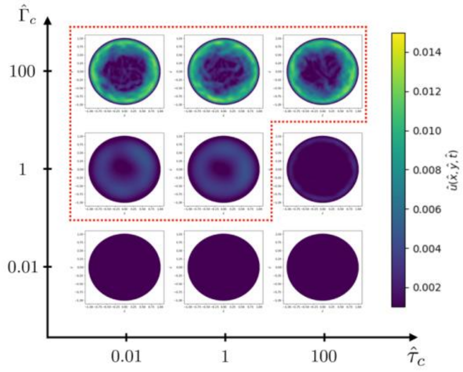

Supplementary Figure 9. Displacement field $\hat{\mathbf{u}}(\hat{\mathbf{x}},\ \hat{t})$ at the time of maximal contraction plotted as a function of $\hat{\Gamma}_c$ and $\hat{\tau}_c$. In all the simulations $\hat{\beta} = 0,\ \hat{\alpha} = 1, \hat{\eta} = 0$ and $c_0 = 1.2$. The red dashed line marks the parameter space for which a global contraction center is formed.

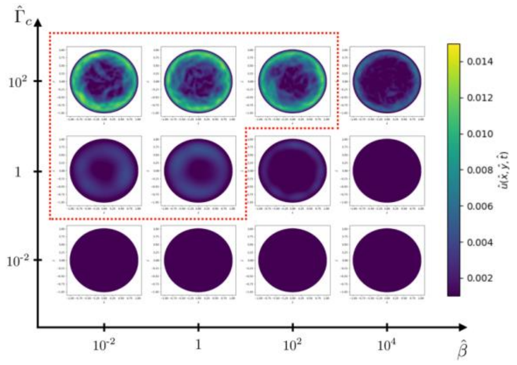

Supplementary Figure 10. Displacement field $\hat{\mathbf{u}}(\hat{\mathbf{x}},\ \hat{t})$ at the time of maximal contraction plotted as a function of $\hat{\Gamma}_c$ and $\hat{\beta}$. In all the simulations $\hat{\alpha} = 1,\ \hat{\tau}_c = 0, \hat{\eta} = 0$ and $c_0 = 1.2$. The red dashed line marks the parameter space for which a global contraction center is formed.

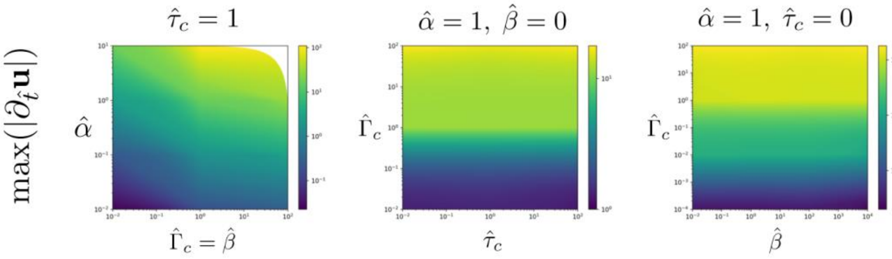

Supplementary Figure 11. Maximum value of the monolayer velocity magnitude, $\max(|\partial_{\hat{t}}\hat{\mathbf{u}}|)$, corresponding to parameter study simulations depicted in Supplementary Figures 8-10.

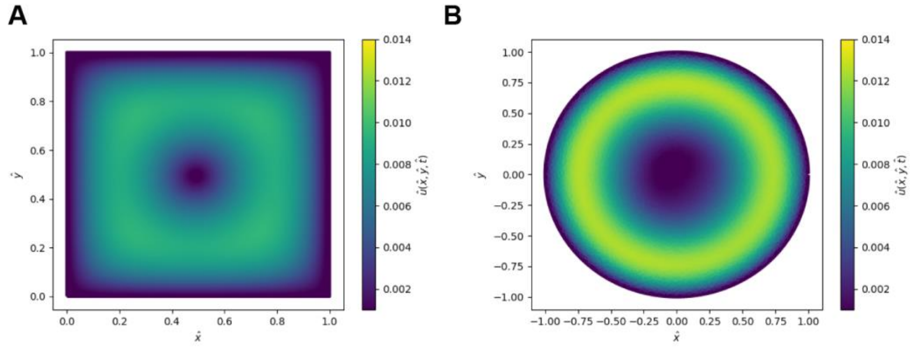

Supplementary Figure 12. Displacement field $\hat{\mathbf{u}}(\hat{\mathbf{x}},\ \hat{t})$ with $\hat{\alpha} = \hat{\beta} = \hat{\tau}_c = \hat{\Gamma}_c = 1$ in a square mesh (A) and a circular mesh (B).

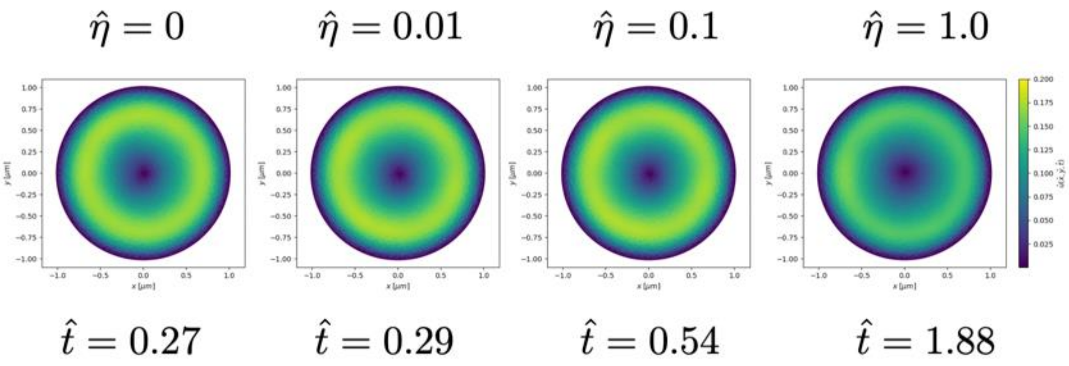

Supplementary Figure 13. Displacement field for four different values of η̂. The time indicates the time of the maximal monolayer contraction. All other non-dimensional parameters are as listed in Supplementary Table 2.

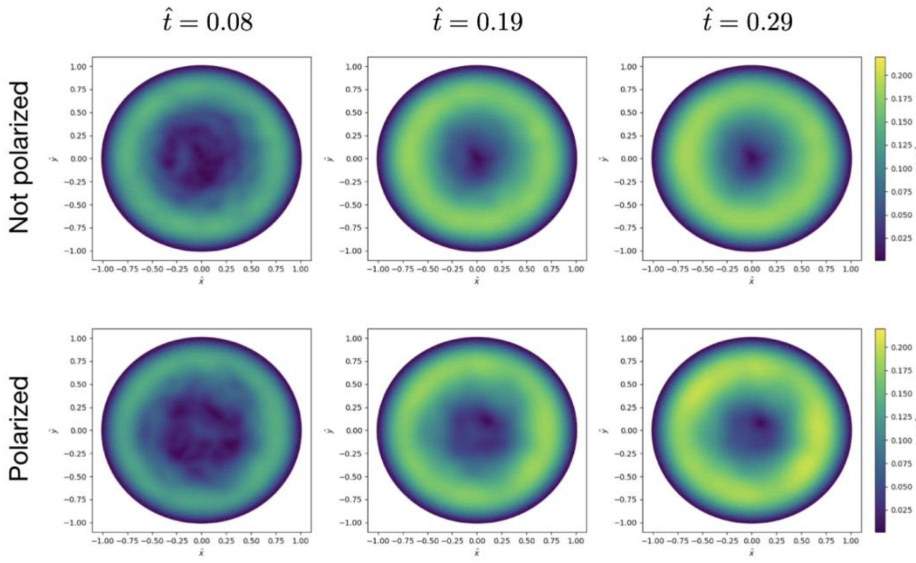

Supplementary Figure 14. Displacement field at three different time points from numerical simulations with a polarization coefficient $\hat{f} = 0$ (top panel), and $\hat{f} = 1.125$ (bottom panel).

Supplementary Videos

Supplementary Video 1

Movies showing cell sheet dynamics in 96-well glass bottom plates. Left panel: proliferation (HaCaT keratinocytes without treatment before imaging); Middle panel: quiescence (HaCaT keratinocytes serum-depleted for 48 h before imaging); Right panel: fluidization (HaCaT keratinocytes serum-depleted for 48 h and subsequently serum re-stimulated before imaging). Microscopy settings: 4x air objective, binning 2 (3.367x3.367 µm pixel size), a time interval of 16 min and an imaging period time of 30 h. The movie covers an imaging period of 27 h and every second frame is shown. Each frame is composed of 4 tiled images. Scale bar, 1 mm.

Supplementary Video 2

Visualization of serum-stimulated cell sheet dynamics based on cell flow (left panel), cell density (middle panel), and cell sheet thickness (right panel). Microscopy settings: 4x air objective, binning 2 (3.367x3.367 µm pixel size), a time interval of 16 min and an imaging period time of 30 h. The movie covers an imaging period of 27 h and every second frame is shown. Each frame is composed of 4 tiled images.

Supplementary Video 3
Basal actin dynamics 0 to 2 h after serum stimulation. HaCaT cells expressing LifeAct-RFP were subjected to serum deprivation for 48 h and subsequently re-stimulated with serum. A single confocal z-plane representing the basal cell surface is shown. The movie shows the two first hours after serum stimulation. The frame interval is set to 2 min between frames.

Supplementary Video 4
Basal actin dynamics 15 to 16 h after serum stimulation. HaCaT cells expressing LifeAct-RFP were subjected to serum deprivation for 48 h. Cells were then re-stimulated with serum for 15 h before imaging. A single confocal z-plane representing the basal cell surface is shown. The movie shows basal actin dynamics between 15 and 16 h after serum re-stimulation. The frame interval is set to 1 min between frames.

Supplementary Video 5
Visualization of local relative cell positioning in a serum-stimulated confluent quiescent cell sheet. HaCaT cells expressing mCherry-Histone H2B were subjected to serum deprivation for 95 h, re-stimulated with serum and the collective cell migration response was monitored by live cell imaging (left panel). The right panel shows a close up on a group of cells, denoted by the white square in the left panel. The cropped frame is generated by image-based registration using a selected cell in the middle of the frame as a tie point. Microscopy settings: 10x FLUAR objective (1331x1331 µm field of view), binning 1, a time interval of 16 min and a total imaging period of 50 h. Scale bar, 10 µm.

Supplementary Video 6
Traction force time lapse microscopy of a serum-stimulated confluent quiescent cell sheet. Phase contrast (left panel), traction forces (middle panel), and intercellular tension (right panel) are depicted. Microscopy settings: 10x FLUAR objective (1331x1331 µm field of view), binning 1. A time interval of 16 min and a total imaging period of 15 h are shown.

Supplementary Video 7
Time lapse of the cellular density in the monolayer obtained from a numerical simulation using a normalized initial concentration $c_0 = 1.002$. We observe that there is a small initial rearrangement in the monolayer density due to the inhomogeneous concentration. Due to the small gradients in the concentration there is no collective motion and the monolayer quickly adopts a quasi-static homogenous profile.

Supplementary Video 8
Time lapse of the cellular density in the monolayer obtained from a numerical simulation

using a normalized initial concentration $c_0 = 1.2$. After the formation of many strong local contraction centers there is a collective cell migration response towards the center of the monolayer. This collective migration results in the formation of one large global contraction center.

Supplementary Video 9

Time lapse of the cellular density in the monolayer obtained from a numerical simulation using a normalized initial concentration $c_0 = 1.09$. At higher concentration levels we observe coordinated cellular motion in the monolayer after the initial formation of small local contraction centers. However, the concentration gradients are not large enough to stimulate the formation of a global contraction center.

References

1. Alert R & Trepat X (2020) Physical models of collective cell migration. *Annual Review of Condensed Matter Physics* 11:77-101.
2. Alt S, Ganguly P, & Salbreux G (2017) Vertex models: from cell mechanics to tissue morphogenesis. *Philosophical Transactions of the Royal Society B: Biological Sciences* 372(1720):20150520.
3. Bi D, Lopez J, Schwarz JM, & Manning ML (2015) A density-independent rigidity transition in biological tissues. *Nature physics* 11(12):1074-1079.
4. Fletcher AG, Osterfield M, Baker RE, & Shvartsman SY (2014) Vertex models of epithelial morphogenesis. *Biophysical journal* 106(11):2291-2304.
5. Banerjee S & Marchetti MC (2019) Continuum Models of Collective Cell Migration. *Advances in experimental medicine and biology* 1146:45-66.
6. Banerjee S, Utuje KJ, & Marchetti MC (2015) Propagating Stress Waves During Epithelial Expansion. *Physical review letters* 114(22):228101.
7. Köpf MH & Pismen LM (2013) A continuum model of epithelial spreading. *Soft matter* 9(14):3727-3734.
8. Loewe B, Serafin F, Shankar S, Bowick MJ, & Marchetti MC (2020) Shape and size changes of adherent elastic epithelia. *Soft Matter* 16(22):5282-5293.
9. Notbohm J*, et al.* (2016) Cellular Contraction and Polarization Drive Collective Cellular Motion. *Biophysical journal* 110(12):2729-2738.
10. Armengol-Collado J-M, Carenza LN, & Giomi L (2022) Hydrodynamics and multiscale order in confluent epithelia. *arXiv preprint arXiv:2202.00651*.
11. Blanch-Mercader C*, et al.* (2017) Effective viscosity and dynamics of spreading epithelia: a solvable model. *Soft Matter* 13(6):1235-1243.
12. Pérez-González C*, et al.* (2019) Active wetting of epithelial tissues. *Nature physics* 15(1):79-88.
13. Larripa K & Mogilner A (2006) Transport of a 1D viscoelastic actin-myosin strip of gel as a model of a crawling cell. *Physica A* 372(1):113-123.
14. Oster GF & Odell GM (1984) Mechanics of cytogels I: oscillations in physarum. *Cell motility* 4(6):469-503.
15. Ronceray P, Broedersz CP, & Lenz M (2016) Fiber networks amplify active stress. *Proceedings of the National Academy of Sciences of the United States of America* 113(11):2827-2832.
16. Banerjee S & Marchetti MC (2019) Continuum models of collective cell migration. *Cell Migrations: Causes and Functions*:45-66.
17. Krajnc M, Dasgupta S, Ziherl P, & Prost J (2018) Fluidization of epithelial sheets by active cell rearrangements. *Physical Review E* 98(2):022409.

18. Ranft J, *et al.* (2010) Fluidization of tissues by cell division and apoptosis. *Proc Natl Acad Sci U S A* 107(49):20863-20868.
19. Boocock D, Hino N, Ruzickova N, Hirashima T, & Hannezo E (2021) Theory of mechanochemical patterning and optimal migration in cell monolayers. *Nature physics* 17(2):267-274.
